# Epiphany: predicting Hi-C contact maps from 1D epigenomic signals

**DOI:** 10.1186/s13059-023-02934-9

**Published:** 2023-06-06

**Authors:** Rui Yang, Arnav Das, Vianne R. Gao, Alireza Karbalayghareh, William S. Noble, Jeffrey A. Bilmes, Christina S. Leslie

**Affiliations:** 1 Memorial Sloan Kettering Cancer Center, New York, USA; 2 University of Washington, Seattle, USA

**Keywords:** 3D genome, Epigenomics, Deep learning

## Abstract

Recent deep learning models that predict the Hi-C contact map from DNA sequence achieve promising accuracy but cannot generalize to new cell types and or even capture differences among training cell types. We propose Epiphany, a neural network to predict cell-type-specific Hi-C contact maps from widely available epigenomic tracks. Epiphany uses bidirectional long short-term memory layers to capture long-range dependencies and optionally a generative adversarial network architecture to encourage contact map realism. Epiphany shows excellent generalization to held-out chromosomes within and across cell types, yields accurate TAD and interaction calls, and predicts structural changes caused by perturbations of epigenomic signals.

**Supplementary Information:**

The online version contains supplementary material available at 10.1186/s13059-023-02934-9.

## Background

In vertebrate genomes, the three-dimensional (3D) hierarchical folding of chromatin in the nucleus plays a critical role in the regulation of gene expression, replication timing, and cellular differentiation [1, 2]. This 3D chromatin architecture has been elucidated through genome-wide chromosome conformation capture (3C) assays such as Hi-C, Micro-C, HiChIP, and ChIA-PET [3–6] followed by next generation sequencing, yielding a contact matrix representation of pairwise chromatin interactions. Early Hi-C analyses revealed an organization of $\sim$1Mb self-interacting topologically associating domains (TADs) that may insulate within-TAD genes from enhancers outside of TAD boundaries [7]. High-resolution 3C-based studies have mapped regulatory interactions, often falling within TADs, that connect regulatory elements to target gene promoters [8, 9].

Over the past decade, large consortium projects as well as individual labs have extensively used 1D epigenomic assays to map regulatory elements and chromatin states across numerous human and mouse cell types. These include methods to identify chromatin accessible regions (DNase I hypersensitive site mapping, ATAC-seq) as well as transcription factor occupancy and histone modifications (ChIP-seq, CUT&RUN). While at least some of these 1D assays have become routine, mapping 3D interactions with Hi-C remains relatively difficult and prohibitively costly, and high-resolution contact maps (5 kb resolution,  2 billion read pairs) are still only available for a small number of cell types. This raises the question of whether it is possible to train a model to accurately predict the Hi-C contact matrix from more easily obtained 1D epigenomic data in a cell-type-specific fashion. Such a model could ultimately be used to predict how perturbations in the 1D epigenome—including deletion of TAD boundaries or inactivation of distal regulatory elements—would impact 3D organization.

Initial machine learning methods to predict Hi-C interactions from 1D epigenomic data or DNA sequence took a pairwise approach, treating each interacting or non-interacting pair of genomic bins as an independent training example [10, 11]. For example, HiC-Reg [10] used a random forest regression model to predict the Hi-C contact signal from epigenomic features of the pair of anchoring genomic intervals. Two more recent models, DeepC [12] and Akita [13], respectively predict ‘stripes’ or submatrices of the Hi-C contact matrix from DNA sequence, capturing the non-independence of interaction bins. Neither method uses epigenomic data as an input signal. DeepC [12] presented a transfer learning framework by pre-training a model to predict epigenomic marks from DNA sequence in order to learn useful local sequence representations, then fine-tuning the model to predict the Hi-C contact map. Akita [13] designed a deep convolutional neural network to predict the Hi-C contact maps of multiple cell types from DNA sequence. These prior studies represent a significant advance in predicting 3D genomic structure, and the DeepC and Akita models demonstrated some success in predicting the impact of sequence perturbations like structural genetic variants on local chromatin folding. However, there are also clear limitations to these approaches. Models that start with DNA sequence need considerable computational resources to extract and propagate useful information from base-pair resolution to megabase scale. More importantly, by learning mappings from only DNA sequence to Hi-C contact map data in the training cell types—and therefore lacking any cell-type-specific feature inputs—the resulting models cannot generalize to new cell types that are not seen in training. In fact, it has also been observed that sequence-based models capture very limited cell-type-specific information about 3D genomic architecture even across the training data and instead predict similar structures in every cell type [13].

Here, we propose a novel neural network model called Epiphany to predict the cell-type-specific Hi-C contact map from five commonly generated epigenomic tracks that are already available for a wide number of cell types and tissues: DNase I hypersensitive sites and CTCF, H3K27ac, H3K27me3, and H3K4me3 ChIP-seq. Epiphany uses 1D convolutional layers to learn local representations from the input tracks as well as bidirectional long short term memory (Bi-LSTM) layers to capture long term dependencies along the epigenome and, optionally, a generative adversarial network (GAN) architecture to encourage realism. One goal of our study is to predict contact maps that are usable for downstream computational analyses such as TAD and interaction calls. To this end, we assessed model performance using multiple normalization and matrix balancing techniques including Knight-Ruiz (KR) [14], iterative correction (ICE) [15], and HiC-DC+ [16] *Z*-score and observed-over-expected count ratio. Epiphany is trained with either MSE alone or with a combination of mean-squared error (MSE) and adversarial loss to enhance its ability to produce realistic rather than highly smoothed Hi-C contact maps. Use of MSE+GAN loss enables improved biological interpretation with only a small trade-off in accuracy for downstream prediction tasks. The adversarial loss is calculated using a simultaneously trained GAN-style discriminator network, which distinguishes real contact maps from predicted ones, and helps the model to improve its prediction quality. Epiphany shows robust performance and generalization abilities to held-out chromosomes within and across cell types and species, and its predicted contact matrices yield accurate TAD and significant interaction calls. At inference time, Epiphany can be used to study the contribution of specific epigenomic signals to 3D architecture and to predict the structural changes caused by perturbations of epigenomic signals.

## Results

### Epiphany: A CNN-LSTM trained with an adversarial loss accurately predicts Hi-C contact maps

Epiphany uses epigenomic signals (DNaseI, CTCF, H3K27ac, H3K27me3, H3K4me3) to predict normalized Hi-C contact maps. Epigenomic signals are extracted at 100bp resolution from normalized .bigWig files without applying a peak calling step. Hi-C contact maps were initially binned at 10 kb resolution and normalized using the HiC-DC+ package [16] to produce *Z*-scores and observed-over-expected (obs/exp) count ratios, Juicer Tools [17] for KR normalization, and HiCExplorer [18] for ICE normalization. The normalization approaches provided by HiC-DC+ are derived from a negative binomial regression that is estimated directly from count data and adjusts for genomic distance and other covariates.

**Fig. 1:**
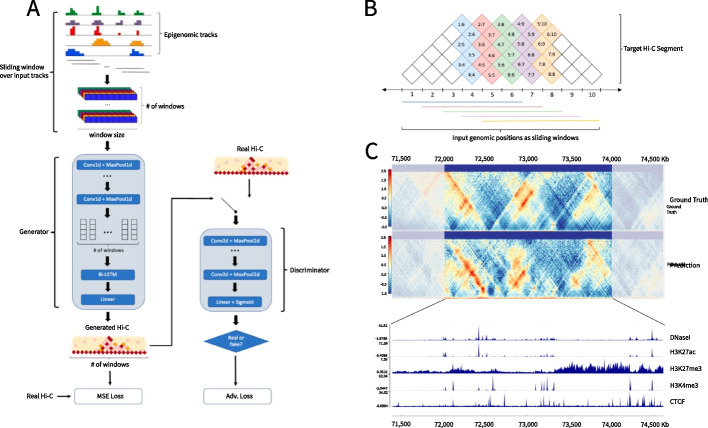
Epiphany employs long short-term memory and adversarial loss to predict the Hi-C contact map. **A** Architecture of Epiphany. Epigenomic signal tracks are first presented to the model in a sliding window fashion, with window size of 1.4 Mb and step size of 10 kb. During training, we take a total length of 3.4 Mb of the input (200 windows) in one pass. In the generator, the processed input data is first featurized by convolution modules, followed by a Bi-LSTM layer to capture the dependencies between nearby bins. After a fully connected layer, the predicted contact map is generated. An MSE loss between the predicted map and the ground truth is calculated in order to train the generator to predict correct structures. To mitigate the overly-smoothed predictions by the pixel-wise losses, we further introduced a discriminator and adversarial loss. The discriminator consists of several convolution modules, and an adversarial loss was calculated to enable the model to generate highly realistic samples. We trained Epiphany with a combined loss of these two components. **B** An illustration of prediction scheme. The first window of input data (blue horizontal line, 1.4 Mb) is used to predict a vector on the Hi-C contact map that is orthogonal to the diagonal (blue bin vector, covers 1 Mb from the diagonal). Note that an extra .2 Mb of input is added to either side of each input window (a total length of 1.4 Mb instead of 1 Mb) in order to provide the model with additional context. During training, 3.4-Mb input tracks are processed using sliding windows (200 windows) in one pass, and 200 consecutive vectors are being predicted. **C** An example region of input epigenomic tracks (bottom), target Hi-C map (top row), and predicted Hi-C map (second row)

Epiphany can be trained with MSE alone or with a combination of MSE and GAN loss. In the latter case, the full model consists of two parts: a generator to extract information and make predictions, and a discriminator to introduce adversarial loss into the training process (Fig. 1A and in the “Methods” section). In the generator, we first used a series of convolution modules to featurize epigenomic information in a sliding window fashion. For one output vector, which covers a distance of 1Mb orthogonal to the diagonal, we used a window size of 1.4 Mb centered at the corresponding region as input (Fig. 1B). Then a Bi-LSTM layer was employed to capture the dependencies between output vectors, so that a total of 3.4 Mb input were processed in one pass for prediction of 200 output vectors. At the end, a fully connected layer was used to integrate signals and make the final prediction. We also introduced an adversarial loss and a discriminator, which consists of several convolution modules that are applied during training and pushes the generator to produce realistic samples (Fig. 1C).

Given the sequential nature of Hi-C contact maps, interactions on consecutive output vectors are unlikely to be independent from one another. We found that Bi-LSTM layers introduce strong dependencies between the output vectors, which allows Epiphany to leverage structures that span multiple genomic positions in Hi-C maps (such as edges of TADs). Furthermore, Bi-LSTM layers overcome the limitation of convolutional neural networks (CNNs) by enabling each output vector to make use of important signals beyond the input window. This is conducive to studying the contribution of distal regulatory elements towards 3D genome structures and reduces the sensitivity of model performance to the choice of window size.

Past approaches that predict the 3D genome structure from 1D inputs use pixel-wise MSE to quantify the similarity between predicted and ground truth Hi-C maps. However, pixel-wise losses for images have been shown by the computer vision community to be overly sensitive to noise [19] and to yield blurry results when used as objectives for image synthesis [20, 21]. In the context of predicting Hi-C maps, MSE loss can over-penalize poor performance on featureless, noisy regions while giving perceptually incorrect regions of significant interactions. These issues can be mitigated with an adversarial loss, which enables the model to generate highly realistic samples while circumventing the need to explicitly define similarity metrics for complex modalities of data. Thus, Epiphany provides the option of training using a convex combination of MSE loss and adversarial loss. A parameter $\lambda$ was introduced to balance the proportion of MSE loss and adversarial loss, and the loss function was defined as

1 $$\begin{aligned} \min _{\theta ^{\mathcal {G}}} \max _{\theta ^{\mathcal {D}}} (1 - \lambda ) \mathcal {L}_{adv}(\theta ^{\mathcal {G}}, \theta ^{\mathcal {D}}) + \lambda \mathcal {L}_{MSE}(\theta ^{\mathcal {G}}) \end{aligned}$$

where $\mathcal {L}_{adv}(\theta ^{\mathcal {G}}, \theta ^{\mathcal {D}})$ is the adversarial loss, and $\mathcal {L}_{MSE}(\theta ^{\mathcal {G}})$ is the MSE between the predicted contact map and ground truth. Intuitively, the MSE loss ensures that the Hi-C maps predicted by Epiphany are aligned with their corresponding epigenomic tracks, while the adversarial loss ensures that the predictions are realistic. We found that using this customized training objective yields realistic Hi-C maps—rather than the smooth and ‘idealized’ maps produced by MSE—that aid in biological interpretation, including after *in silico* epigenomic perturbation experiments. However, we found that both the MSE and MSE+GAN versions of Epiphany produced maps that can be directly processed by commonly used downstream analysis tools for TAD and interaction calls.

### Training with MSE alone leads to prediction of perceptually poor contact maps

To illustrate why MSE alone can be an inadequate metric for reconstructing and evaluating the quality of the predicted contact map, we conducted two analyses: first, we examined how synthetic contact maps could achieve good MSE while failing to capture features of true Hi-C maps; and second, we considered the spatial properties of predicted regions of significant interactions in the MSE-only and MSE+GAN models. Additional file 1: Fig. S1 shows true, predicted and synthetic Hi-C maps for three example regions on chromosome 3. Each Hi-C map is rotated: the y-axis on the left shows the genomic coordinates, and the *x*-axis shows the distance from the diagonal. From left to right we plot: the ground truth Hi-C map, the prediction from MSE model, the prediction from GAN model, synthetic Hi-C map 1 (local average of the Hi-C map), synthetic Hi-C map 2 (local average for each genomic distance on the Hi-C map), synthetic Hi-C map 3 (averaged value at each genomic distance). The MSE value compared with ground truth and the discriminator score calculated from the well-trained discriminator are shown in the subtitles. Although the synthetic maps look nothing like the real Hi-C map, they can still achieve a low MSE score compared to ground truth. For example, synthetic map 1 has equivalent MSE loss compared to the GAN prediction; synthetic map 2 has almost the same MSE loss as the blurry prediction. We conclude that MSE alone does not capture the quality of the contact map, and that optimizing MSE alone may not be enough to generate good predictions. We therefore added a GAN component to push the model to generate more realistic maps. The ‘Disc’ value indicates the score calculated from the well-trained discriminator, where a high score means the matrix is very Hi-C-like, and a low score indicates the matrix is unlike true Hi-C. The discriminator can easily tell that the synthetic matrices are not Hi-C matrices.

To further explore the nature of the ‘blurry’ MSE-only model predictions, we examined the predicted regions of significant interactions in each case. In Additional file 1: Fig. S2, we extracted random regions from test chromosome 3 of GM12878 (size: 2Mb along the diagonal, 1Mb from the diagonal). In each case, the left column shows the Hi-C map of ground truth (top), prediction from the MSE-only trained model (middle), and prediction from the MSE+GAN model (bottom), using O/E target values. The middle column shows the significant interactions called from each map, where interactions $>= 2$ are marked as 1 (“significant”), and interactions $< 2$ are marked as 0 (“not significant”). The right column shows the absolute difference of the significance plot (green = 0, no difference; yellow = 1, false negatives, purple = − 1, false positives). In each case, the MSE-only blurry prediction gives slightly better accuracy than the GAN prediction. However, if we check the spatial distribution of the significant calls, the GAN model produces better structure than the MSE-only model. We can see from the binarized interaction plot (middle column) that predictions from the MSE-only model give “blob-like” regions of significant interactions due to its blurriness, while the GAN model identifies edges and TAD-like structures. We also found that the false predictions from the GAN model were usually caused by a small pixel shift.

The observations above that the MSE relative to ground truth can be good even for a perceptually poor synthetic or predicted contact map (Additional file 1: Fig S1) and the perceptually apparent structural problems of the MSE-only binarized predictions (Additional file 1: Fig. S2) are related to the “perception-distortion trade-off” that has been described theoretically in the image reconstruction literature [22], where the model tries to reconstruct an image $\hat{X}$ from a degraded version of the original image *X*. Briefly, the authors show that there is a trade-off between optimizing the distortion measure between the reconstructed image $\hat{X}$ and original image *X* vs. improving the perceptual quality of $\hat{X}$, and a GAN provides a principled approach to navigate this trade-off.

When we train Epiphany with both MSE and adversarial loss, we can use the well-trained discriminator from the model as a perceptual score to evaluate predictions. In Additional file 1: Fig. S3, we plot the perceptual score for all sub-regions (200$\times$100, 2Mb along the diagonal, 1M from the diagonal) predicted on chr3. In Additional file 1: Fig. S3A, the *x*-axis shows the genomic location of each 2Mb$\times$1Mb sub-region along the diagonal and the *y*-axis shows the perceptual score. Blue dots show the perceptual score for ground truth Hi-C, green dots for the GAN prediction, and orange for the MSE-only prediction. All contact maps predicted by the MSE-only model obtain very low perceptual scores. In Additional file 1: Fig. S3B, we show example regions with the highest perceptual score in ground truth (a), MSE-only prediction (b), and GAN prediction (c); note that these examples correspond to different genomic locations.

### Epiphany accurately predicts the Hi-C contact map

**Fig. 2:**
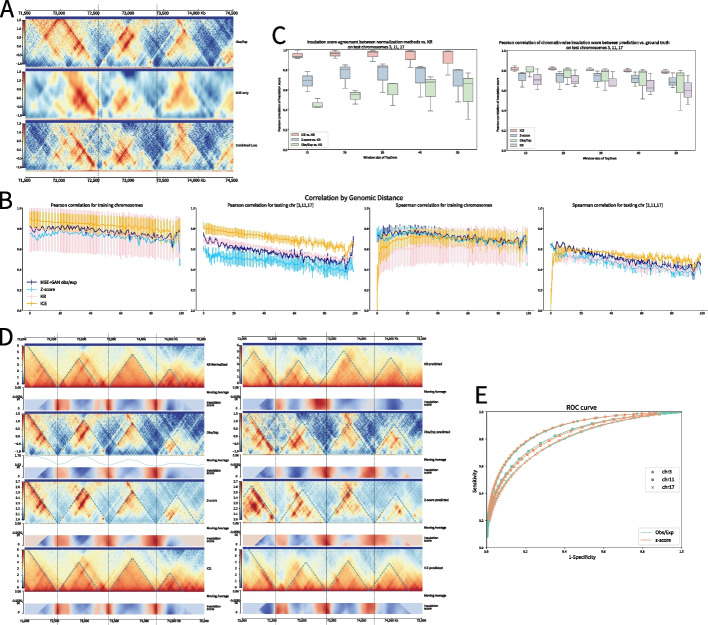
Epiphany-predicted contact maps identify TADs and significant interactions. **A** A visual comparison of the ground truth contact map (chr17:70,670,000–73,880,000, top row), blurry prediction made by MSE trained model (middle row), and more realistic prediction by combined loss (bottom row). **B** Epiphany performance using correlation by genomic distance for different normalization approaches. From left to right: Pearson correlation on training chromosomes, on testing chromosomes (chr3, 11, 17), and Spearman correlation on training and on testing chromosomes. Dark blue shows the performance of HiC-DC+ observed-over-expected count ratio, light blue shows HiC-DC+ *Z*-score, pink shows KR normalization, and yellow shows ICE normalization. **C** Left: Agreement of insulation score between different normalization methods vs. KR normalization on test chromosomes. Insulation scores were calculated using TopDom with different window sizes (*X*-axis) on ground truth contact maps with different normalization methods. KR normalization was used as the gold standard, and a Pearson correlation (*Y*-axis) was calculated to measure the agreement between each normalization method vs. KR (red: ICE vs. KR, blue: HiC-DC+ *Z*-score vs. KR, green: HiC-DC+ obs/exp vs. KR). Right: Pearson correlation of insulation score between predicted contact map vs. corresponding ground truth of the same normalization (red: ICE, blue: HiC-DC+ *Z*-score, green: HiC-DC+ obs/exp, purple: KR). **D** Left: Ground truth contact maps of different normalization methods (from top to bottom: ICE, HiC-DC+ obs/exp, HiC-DC+ *Z*-score, KR). Blue dashed lines denotes the TAD calls with window size of 50 on each contact map, and black dashed lines are the TAD boundaries called from KR normalized contact map. Right: Predicted contact maps of different normalization. **E** ROC curve of significant interactions between prediction contact maps vs. ground truth for the three test chromosomes for HiC-DC+ obs/exp ratios (green) and for *Z*-scores (orange)

We first benchmarked the model at 10 kb resolution to compare between two loss functions: MSE only and the convex combination of MSE and adversarial loss. Both losses use the observed-over-expected count ratio normalization based on HiC-DC+. Models were trained on data from the GM12878 ENCODE cell line, with chr3, 11, and 17 as completely held-out chromosomes. Epiphany demonstrates good performance for both the Pearson and Spearman correlation metrics using the observed-over-expected count ratio (Table 1), while MSE produced higher correlations than the convex combination of MSE and adversarial loss. However, as discussed above, we observed that the high correlations from MSE trained models were associated with blurriness and poorer perceptual quality in the predicted contact maps (Fig. 2A), whereas the correlations produced by the combined loss models may have been slightly diminished due to small deviations in the sharper predictions. Therefore, we reasoned that correlation may not be an appropriate evaluation metric and decided to use the combined loss (MSE+adversarial loss) for most of the downstream analyses below; however, users can choose between the MSE-only and MSE+GAN training modes depending on the desired trade-off between higher correlation vs. better perceptual quality for their application.

**Table 1:** Mean Pearson and Spearman correlation for different normalization methods

Normalization Method	$\lambda$	Pearson (all)	Pearson (train)	Pearson (test)	Spearman (all)	Spearman (train)	Spearman (test)
Obs/Exp	0.95	0.7408	0.7687	0.5636	0.6899	0.7191	0.5048
Obs/Exp	1 (MSE only)	0.7833	0.8045	0.6494	0.7381	0.7605	0.5963
*Z*-score	0.95	0.6881	0.7222	0.4722	0.6695	0.7034	0.4544
KR	0.35	0.7289	0.7510	0.5889	0.5909	0.6135	0.4477
ICE	0.35	0.8108	0.8288	0.7028	0.6631	0.6852	0.5303

We next tested the robustness of Epiphany with various normalization methods, including KR normalization, ICE normalization, and obs/exp values and *Z*-scores from HiC-DC+. All models were set up with the same training approach as before, where chr3, 11, and 17 were used as held-out chromosomes and models were trained with the combined loss. Epiphany shows robust performance in all normalization methods (Fig. 2B). ICE normalization obtained the highest correlations, with an average Pearson correlation of 0.7028 and Spearman correlation of 0.5303 on completely held-out chromosomes (Table 1). We observed a correlation spike towards the end of the genomic distance range, apparently caused by an edge effect artifact in prediction of the contact map. In practice, we suggest that a smaller region (strictly smaller than 1Mb from the diagonal) of the predicted contact map should be used in downstream analyses to avoid this edge effect, or the model architecture should be slightly altered to predict a wider ($>1$Mb from the diagonal) region to push the edge beyond the desired field of view.

To explore the capacity of Epiphany to capture key structures in genome architecture, we next evaluated the ability of Epiphany predictions to recover TAD boundaries. For all normalization methods and their predictions, we called TAD boundaries using TopDom [23] with window sizes ranging from 10 to 50 (corresponding to 100 kb to 500 kb regions). Because TAD calls depend on the normalization method, we first used KR normalization as the gold standard and compared TAD insulation scores computed on ground truth data on the test chromosomes using different normalization methods. Note that we chose to compare insulation scores rather than TAD boundaries, since the latter relies on finding local extrema in the insulation score signal and therefore can be unstable. Among all these methods, ICE had the highest consistency with KR, followed by *Z*-scores calculated from HiC-DC+. The observed-over-expected count ratios had the least consistency and showed large variation over the three test chromosomes (Fig. 2C, left). We then compared the insulation score calculated from the Epiphany-predicted contact maps trained with different normalization methods vs. the corresponding ground truth on the test chromosomes. ICE showed robust predictions on all test chromosomes, whereas HiC-DC+ observed-over-expected count ratio normalization displayed strong mean performance but had larger variance, especially for larger window sizes. HiC-DC+ *Z*-scores and KR normalization showed lower consistency between predicted vs. ground truth insulation scores (Fig. 2C, right). From a visual comparison of ground truth and predicted contact maps with different normalization approaches, we can see Epiphany consistently predicts accurate TAD structures (Fig. 2D, epigenomic tracks for these loci at Additional file 1: Fig. S4). Overall, this analysis suggests that, for accurate prediction of TAD structure, Epiphany trained on ICE normalized contact maps gave the best performance, with HiC-DC+ observed-over-expected count ratio as runner-up.

One advantage of HiC-DC+ normalization is that it readily allows the comparison of significant interactions between predicted contact maps and ground truth. HiC-DC+ [16] fits a negative binomial regression using genomic distance, GC content, mappability and effective bin size based on restriction enzyme sites to estimate the expected read count for each interaction bin, which allows an assessment of significance of the observed count. For convenience, we defined the significant interactions as ground truth *Z*-scores greater than 2. Significant interactions were called with various thresholds from test chromosomes on predicted contact maps using *Z*-scores and observed-over-expected count ratio, yielding the ROC curves for each test chromosome (Fig. 2E). The average AUC is 0.7639 for the two models, suggesting solid performance at a difficult task.

We also compared the TAD insulation score and significant interactions between the MSE-only model and MSE+GAN model using the methods above, and the results are shown in Additional file 1: Fig. S5, S6. For the insulation score comparison, we found that both MSE-only and MSE+GAN perform similarly, since the insulation score used for TAD boundary detection is a sum of a large number of “pixels” from the contact map; however, the MSE-only model produced insulation scores with slightly higher correlation to ground truth (Additional file 1: Fig. S5). For the significant interactions, we defined true significance as ground HiC-DC+ *Z*-score $>= 3$, and found that both models performed well at this task, with the MSE-only slightly outperforming the MSE+GAN model (Additional file 1: Fig. S6, auROC $= 0.8858$ vs. 0.8658, auPR $= 0.4143$ vs. 0.3110). The overall advantage of the MSE-only model over the MSE+GAN model is due to somewhat superior performance for predicting interactions at greater genomic distances (over 250Kb), where true positives are sparse and the smoothed MSE-only predicted contact map has greater recall. Thus, the MSE+GAN model retains strong performance at downstream tasks such as significant interaction and TAD calling, only slightly underperforming the MSE-only model, while achieving perceptually much better predicted contact maps.

### Epiphany shows robust performance at finer resolution

**Fig. 3:**
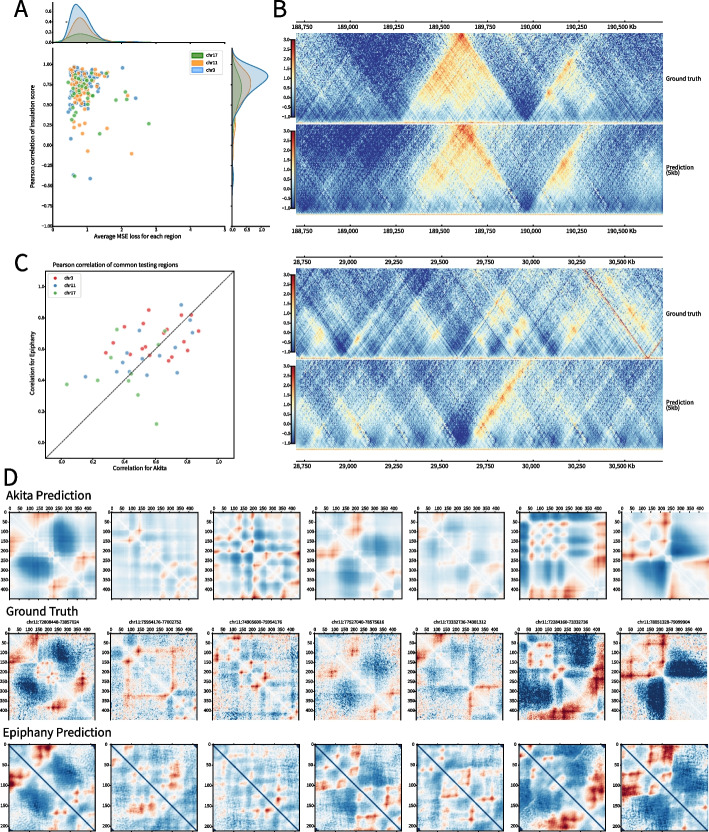
Epiphany achieves state-of-the-art performance at fine resolution. **A** Evaluation of predicted submatrices (2Mb along the diagonal by 1Mb from the diagonal). *X*-axis denotes the average MSE loss between predicted matrix and ground truth, and *Y*-axis shows the Pearson correlation of insulation score of the 2 Mb region. Dots are colored by chromosomes, and density plots for dot distribution are added on the side. **B** Epiphany performance evaluation at 5 kb resolution. Top: one of the best predicted submatrices (chr3:188,610,000–190,610,000) with ground truth matrix on the top, and predicted matrix on the bottom. Bottom: one of the problematic matrices (chr17:28,705,000–30,705,000) predicted by Epiphany. **C** Model performance comparison between Epiphany and Akita on 42 common regions between Akita held-out test regions and our test chromosomes (chr3, 11, 17). *X*-axis shows the Pearson correlation of Akita prediction vs. ground truth, and *Y*-axis shows the correlation of Epiphany. Epiphany was re-trained using data with the same normalization steps of Akita at 5 kb resolution, and Akita predictions were average-pooled into 4096 bp resolution for better comparison. Dots are colored by chromosomes. **D** Visual comparison of Akita prediction (2048 bp resolution, top row), ground truth matrices (2048 bp resolution, middle row), and Epiphany prediction (5 kb, bottom row)

Due to good overall performance and the ability to directly identify significant interactions, we chose observed-over-expected count ratios rather than *Z*-scores from HiC-DC+ for further analysis. We again trained Epiphany to predict interactions within 1Mb from the diagonal at 5 kb resolution. Epiphany showed robust performance at 5 kb resolution, with an average Pearson correlation of 0.5625 and Spearman correlation of 0.5270. In addition to the distance-dependent correlations, we also used both MSE loss and insulation scores calculated from HiCExplorer [18] to evaluate model performance. Since Epiphany jointly predicts multiple interaction vectors, the model can predict a submatrix of the contact map that covers a 2Mb distance along the diagonal (400 vectors for 5 kb resolution) and up to 1Mb from the diagonal. We calculated the average MSE loss between the predicted submatrix vs. ground truth as well as Pearson correlation between insulation scores calculated from the corresponding submatrices. Results for all 2Mb submatrices from the three held-out chromosomes (chr3, 11, 17, Fig. 3A) show that Epiphany displays consistent prediction performance across held-out chromosomes with diverse length and gene densities. In particular, 84.4% (173 out of 203) of submatrices have insulation correlation higher than 0.50. Epiphany showed robust performance in most regions along the genome but sometimes produced inaccurate predictions at regions without clear signals or in low mappability regions. (Fig. 3B).

We also compared Epiphany with Akita [13] on common test regions, restricting our evaluation to regions that were held out by Akita and overlap with our test chromosomes. We binned the Hi-C contact map at 5 kb resolution and followed the normalization approaches suggested in the Akita study (Data sources and pre-processing). Epiphany was re-trained using the training chromosomes as before (all chromosomes except for chr3, 11, 17) and evaluated on the 42 test regions from Akita’s held-out set falling in our test chromosomes. Akita’s predictions at 2048 bp resolution were average-pooled to 4096 bp in order to obtain relatively consistent resolution. For each test region, we calculated the Pearson correlation between predicted contact matrices and ground truth for both Akita and Epiphany (Fig. 3C). We also visually compared the predictions of Akita and Epiphany with ground truth on the held-out examples (Fig. 3D). Quantitatively and qualitatively, both models showed similar performance.

### Epiphany predicts cell-type-specific 3D structure

**Fig. 4:**
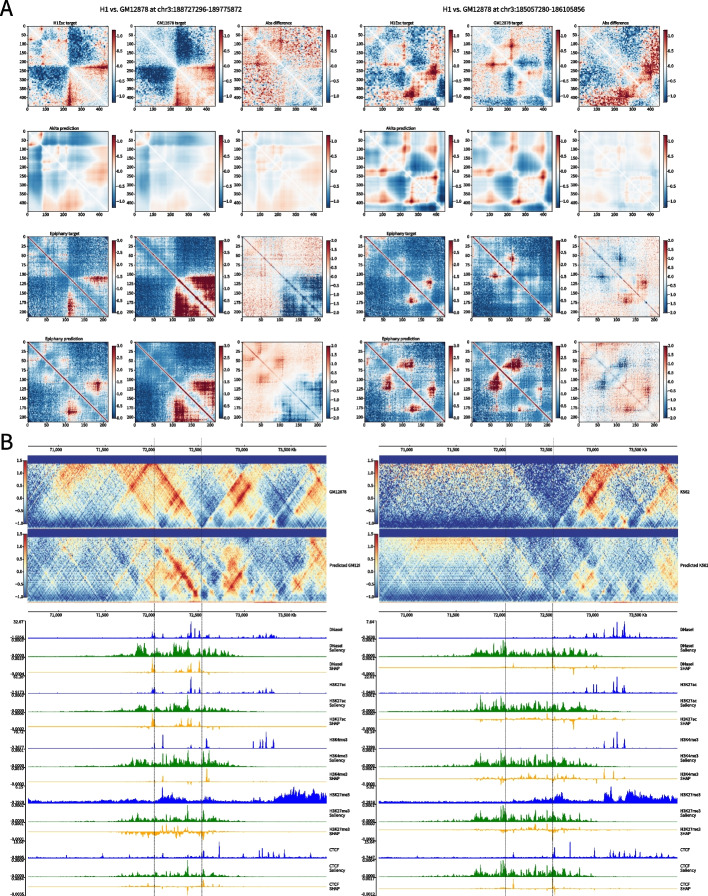
Epiphany accurately predicts cell-type-specific 3D structures. **A** Two examples (chr3:188,727,296–189,775,872) and (chr3:185,057,280–186,105,856) of cell-type specific predictions in H1-hESC and GM12878. Two regions are selected from the overlapped region of Akita held-out test set and Epiphany’s test chromosomes. Columns from left to right: contact map in H1ESc, same region in GM12878, and the absolute difference between the two cell types (H1-GM12878). Rows from top to bottom: Ground truth matrices with Akita normalization, Akita prediction, ground truth with HiC-DC+ observed-over-expected count ratio, Epiphany prediction of observed-over-expected count ratio. Akita predictions were obtained from the multi-task output, and Epiphany predictions were generated with model trained on GM12878. **B** Cell type specific prediction at a differential region between GM12878 and K562. On the left is the ground truth matrix (top) and predicted matrix (middle), followed by epigenomic input tracks (blue), Saliency score (green), and SHAP values (yellow) for feature attributions. On the right is the predictions for K562. Epiphany was trained in GM12878 in training chromosomes, and predicted both cell types for test chromosomes

Since Epiphany uses epigenomic marks as input, it can potentially generalize to predict cell-type-specific 3D structures in a new cell type. We first compared Epiphany’s cell-type-specific predictions with those of Akita, where five different cell types were simultaneously predicted in a multi-task framework (Table 2 and 3). We selected H1-hESC and GM12878 from these five cell types for the comparison. Akita’s cell-type-specific predictions were directly obtained from its multi-task output. Epiphany was trained on GM12878 and evaluated on H1-hESC test chromosomes (chr3, 11, 17) at inference time. We checked the visual comparison of Akita and Epiphany cell-type-specific predictions relative to their respective ground truths and also calculated the absolute difference for ground truth and predictions between the two cell types (Fig. 4A). The results suggest that Epiphany, which was trained only on GM12878 data, can generalize to a new cell type and accurately predict the differential structure between cell types based on cell-type-specific 1D epigenomic data. By contrast, the DNA-sequence-based Akita model, although trained on Hi-C/Micro-C data in these and other cell types, largely predicts the same 3D structure in GM12878 and H1-hESC.

**Table 2:** Hi-C data source

Cell type	Hi-C link
GM12878	4DNFI1UEG1HD
H1-hESC	4DNFIQYQWPF5
K562	4DNFITUOMFUQ
mESC	4DNFI8KBXYNL
HCT116	4DNFIXTAS6EE
HLV (HiCAR)	ENCFF294GFP

**Table 3:** Epigenomic data source

Cell type	Dnase I	CTCF	H3K27ac	H3K27me3	H3K4me3
GM12878	ENCSR000EMT	ENCSR000DRZ	ENCSR000DRY	ENCSR000DRX	ENCSR000AKC
H1-hESC	ENCSR000EMU	ENCSR000AMF	ENCSR000ANP	ENCSR216OGD	ENCSR019SQX
K562	ENCSR000EOT	ENCSR000DWE	ENCSR000AKP	ENCSR000AKQ	ENCSR000DWD
HCT116	ENCSR000ENM	ENCSR000DTO	ENCSR661KMA	ENCSR810BDB	ENCSR333OPW
HLV	ENCSR222CLC	ENCSR461HOC	ENCSR748OJA	ENCSR072GNA	ENCSR359HLC

We also conducted a systematic analyses across all the held-out test regions of the Akita model by performing a cell-type specific comparison with consistent targets (Akita’s normalization method). The results are shown in Additional file 1: Fig. S7, which provides the Pearson correlation of 42 test regions of GM12878 (left) and H1-hESC (right). A full table of Pearson correlation and *p*-values can be find in Additional file 2: Table S1. The *x*-axis shows the correlation between ground truth and prediction using the Akita model, and the *y*-axis shows the correlation using Epiphany. For H1-specific predictions—to provide a fair comparison with Akita—we retrained the Epiphany model using the training chromosomes in H1-hESC, so that the Epiphany predictions in both figures are cross-chromosomal but within cell type. (Due to the sparseness of the ground truth in H1-hESC data, the H1 model was trained with MSE-only loss; in general, we expect that the GAN component of the model is less useful when training on noisier Hi-C data). We found that Epiphany showed equivalent performance to Akita on GM12878 (*p*-value of 0.7279, Wilcoxon signed-rank test) and slightly better than Akita on H1-hESC (*p*-value of 0.0874).

In addition to the comparison with Akita with its pre-trained cell types, we also systematically evaluated Epiphany’s ability to make cell-type-specific prediction in K562. Here, Epiphany was trained on GM12878 and evaluated on the test chromosomes (chr 3, 11, 17) in K562. Similar to the experiment in Fig. 3A and B, the Pearson correlation of insulation scores and average MSE loss were calculated for each 2Mb submatrix. Epiphany showed robust prediction performance when generalizing to a new cell type (Additional file 1: Fig. S8). As a second example beyond cell line data, we also ran a similar analysis to test Epiphany’s ability to make cell-type-specific predictions in a tissue setting. Here Epiphany was trained on GM12878 and tested on left heart ventricle (69 year old male) using intact Hi-C from ENCODE. The results are shown in (Additional file 1: Fig. S9) and demonstrate similar generalization performance in this tissue context as in the cell line test data.

To evaluate Epiphany’s ability to generalize to unseen cell types against currently available methods, we again used Akita as a comparison. Since Akita has not been trained on K562, we averaged the predictions for five pre-trained cell types (GM12878, H1-hESC, HFF, IMR90, HCT116) and checked non-conserved examples from Akita’s 42 test regions. The results are shown in (Additional file 1: Fig. S10). For most of the non-conserved regions, Epiphany can predict the unique structures of the unseen cell type.

We further compared Epiphany’s generalization performance and cell-type-specificity on K562 and H1-hESC. We used the Epiphany model trained on GM12878 training chromosomes (all chromosomes except for 3, 11, 17) and compared its performance on H1-hESC and K562 chromosome 3. We again chopped the chromosome into 2Mb regions along the diagonal, and calculated the Pearson correlation of insulation scores between H1-hESC and K562 from ground truth contact maps for each cell type. We then calculated the Pearson correlation of insulation scores from the predicted maps of the two cell lines. The results are shown in Additional file 1: Fig. S11A, where we plot the Pearson correlation of insulation scores between H1-hESC vs. K562 based on ground truth contact maps (*x*-axis) vs. predicted contact maps (*y*-axis). We then ranked the 2Mb regions based on their insulation score correlation and checked several cell-type-specific predictions visually. Two cell-type-specific regions (top: chr3:78,705,000–80,705,000 and bottom: chr3:705,000–2,705,000) are shown in Additional file 1: Fig. S11B and C: on the top are the ground truth contact maps for K562 and H1-hESC, and at the bottom are the predicted contact maps for the two cell lines. An additional comparison of a cell-type-specific 3D structure region (chr3:188,250,000–190,250,000) in GM12878, K562 and H1-hESC is shown in Additional file 1: Fig. S11D and E. Ground truth Hi-C maps are shown on the left, and Epiphany predictions are on the right.

We next found another example of a cell-type-specific region around the gene CPED1 [24], and we tested whether Epiphany could predict the difference. The CPED1 locus is covered by an H3K27me3 rich region in GM12878 but overlaps with a super-enhancer in K562. Additional file 1: Fig. S11F demonstrates the cell-type-specific prediction performance of Epiphany: (top to bottom) GM12878 ground truth structure; GM12878 contact map predicted by Epiphany; K562 ground truth; K562 contact map predicted by Epiphany; gene annotations; and epigenomic tracks (H3K27ac, H3K27me3) for the two cell types.

We also examined cell-type-specific predictions between K562 and HCT116. The Epiphany model was trained on GM12878 and tested on both K562 and HCT116. We first used HiCExplorer [18] to identify differential TADs on test chromosome 11 between the two cell types and compared predictions from the Epiphany model. Differential regions were identified by Wilcoxon rank-sum test under the null hypothesis that the two cell types were identical, then ranked by the inter-TAD *p*-values. Full tables of conserved and non-conserved regions are attached as Additional file 3: Table S2 and Additional file 4: Table S3. Additional file 1: Fig. S12A shows the distribution of Pearson correlations between prediction vs. ground truth for the top 25 conserved regions (blue) and non-conserved regions (orange) for K562 (left) and HCT116 (right). Epiphany achieves similar performance for cell-type consistent and differential regions. Additional file 1: Fig. S12B shows visual examples of Epiphany’s prediction on the two cell types in the cell-type-specific regions, and Additional file 1: Fig. S12C shows conserved regions. In these examples, Epiphany accurately predicts both conserved and non-conserved structures between cell types.

Finally, we explored the ability of Epiphany to identify the contribution of cell-type-specific epigenomic input features to differential 3D structures using feature attribution. In recent years, feature attribution has become a powerful tool to study the contribution of input features to prediction of a specific output. For each interaction bin in the predicted contact map, we first calculated the saliency score [25], which is a gradient-based attribution on input values. We then calculated the SHAP value [26] with baseline signals equal to zero, which highlights the contribution of epigenomic peaks to a specific output. We compared a region (chr17:70,500,000–73,500,000) with differential interactions between GM12878 and K562 (Fig. 4B). Epigenomic signals between chr17:72,000,000–72,500,000 in GM12878 contributed to the prediction of the highlighted interaction, while the absence of signals in K562 input led to the correct prediction of a weak interaction.

### Ablation analyses suggests redundancies between 1D inputs

In the previous cell-type-specific analysis, distal H3K4me3 peaks gained importance in the K562 prediction when there were no signals at the anchors of the investigated interaction (Fig. 4B). We wondered whether features from different epigenomic tracks could compensate for each other in predicting interactions and more generally whether there exist redundancies between the input tracks.

We performed a feature ablation experiment to address these questions. Instead of including all five epigenomic tracks as input, we re-trained the model with one or several of the tracks completely masked as zero. We reasoned that re-training the model rather than masking a specific input region at test time could better serve our goal. For example, using a model trained on all five input tracks, if we simply masked one important peak from DNaseI track during test time, we expected that the model would inevitably fail to predict the corresponding interactions. However, if we re-trained the model with the entire DNaseI track masked, we expected the model to identify alternative signals from other tracks during training and potentially retain the ability to predict these interactions.

**Fig. 5:**
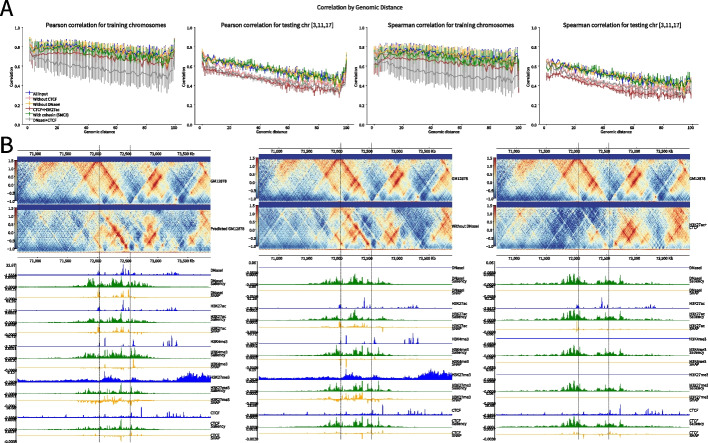
Feature ablation and attribution identify the contribution of epigenomic marks to 3D structure. **A** Correlation by distance for feature ablation experiments. Left to right: Pearson correlation for training chromosomes, Pearson correlation for test chromosomes. Spearman correlation for training chromosomes, Spearman correlation for test chromosomes. Blue track for model performance using all 5 epigenomic input tracks; green for training with an additional track SMC3; pink for model trained without CTCF; yellow for without DNaseI, dark red for only CTCF and H3K27ac tracks, and gray for model trained with only DNaseI and CTCF. **B** Feature attribution for bin (chr17:57,140,000–57,750,000) with Epiphany with Bi-LSTM layer (left) vs. modified Epiphany with convolution layer (right)

Indeed, this idea matched our observations from the ablation analysis. We re-trained Epiphany with (a) an additional SMC3 ChIP-seq track, (b) CTCF track masked as zero, (c) DNaseI track masked as zero, and (d) only CTCF and H3K27ac tracks as training inputs and compared their predictions with the results using all input (Fig. 5A). We found that by removing DNaseI, the model achieved similar performance as using all input tracks. Models with CTCF masked or using only two tracks (CTCF+H3K27ac) showed weaker performance.

As we have seen in previous example (Fig. 4B), DNaseI and H3K27ac contributed to the differential predictions between GM12878 and K562 at the region chr17:70,670,000–73,880,000. We therefore compared the prediction for this region using a model trained with all input tracks, without DNaseI, or with CTCF+H3K27ac only (Fig. 5B). Epiphany was still able to accurately predict interactions in this region after ablating DNaseI; feature attribution indicated that in place of the DNaseI signal (Fig. 5B, grey box), the model gave higher importance to H3K27me3 peaks (purple box) in order to predict the interaction. However, after ablating all signals except for CTCF and H3K27ac, the model failed to find alternative predictive signals and missed the boundary.

A recent manuscript named C.Origami [27] uses DNA sequence as well as ATAC-seq and CTCF tracks for cell-type-specific predictions. From our experiments above, we suggest that there may be redundancies between the information from chromatin accessibility and histone modifications. Therefore, we re-trained Epiphany with only DNaseI and CTCF tracks to test our hypothesis. Consistent with C.Origami, we observed fairly good performance of Epiphany using only DNaseI and CTCF tracks. The predictions have an average Pearson correlation of 0.5789 on the training chromosomes, 0.4638 on the testing chromosomes, and Spearman correlation of 0.5292 and 0.4224 on the training and test chromosomes respectively. The distance-adjusted Pearson and Spearman correlation values are shown in Fig. 5A. A visual comparison of a random region on the test chromosome (chr3:123,675,000–126,675,000) is shown in Additional file 1: Fig. S13.

### Epiphany predicts perturbations in 3D architecture

**Fig. 6:**
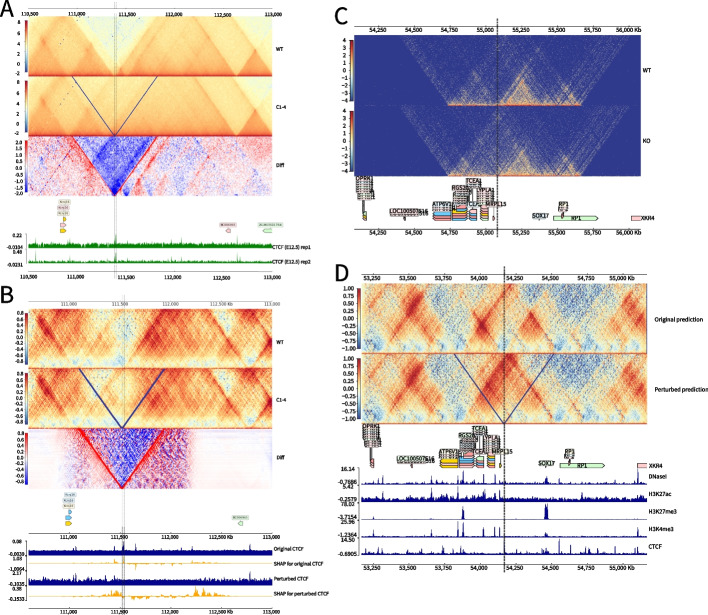
Epiphany predicts TAD boundary changes due to epigenomic perturbations. **A** Mouse ES E12.5 Capture Hi-C data from Despang et al. [28] for WT (top), 4 CTCF sites depletion (middle), and the absolute difference between the two conditions (bottom). Data are publicly available at (GSE78109, GSE125294). Four CTCF sites depleted in the middle figure were at region (C1 site(mm9 chr11:111,384,818–111,385,832), C2-C4 site (chr11:111,393,908–111,399,229), marked with black dashed lines). Data are mapped relative to mm9. **B** Epiphany cross-species prediction of structural changes caused by CTCF perturbation. Epiphany was trained using human cell line GM12878, and predicted using mouse limb bud epigenomic data mapped relative to the mm10 assembly. The panel shows Epiphany prediction of WT mES Hi-C map with HiC-DC+ obs/exp ratio normalization (top row), the prediction of TAD fusion after masking CTCF sites at (mm10 chr11:111,520,000–111,540,000) (middle row), and the absolute difference between the two predictions (bottom row). Epigenomic tracks at the bottom are showing feature attribution (SHAP value) for highlighted vertical vector in the Hi-C contact map. The upper two tracks are the original CTCF track and corresponding SHAP values. The lower two tracks are CTCF tracks with peaks in the vertical line deleted and the corresponding SHAP values. **C** Capture Hi-C maps from Wu et al. [31] experimented at the *Sox17* locus before (top) and after (bottom) targeting the CTCF sites. Data are publicly available at (GSE127196). Data are mapped relative to hg19. **D** Epiphany cross-cell type prediction of structural changed caused by CTCF deletion. CTCF sites at hg38 (chr8:54,165,000–54,170,000, in the vertical lines) near *Sox17* locus is deleted. Top row shows the Epiphany predicted Hi-C contact map before perturbation, and bottom row shows the predicted map after deletion. Vertical dashed line shows the location of the deleted CTCF site

Since Epiphany models the contribution of epigenomic signals to 3D structures, we explored whether Epiphany could predict 3D structural changes caused by perturbations to the epigenome. In particular, we considered examples where structural variations eliminate important epigenomic features. Despang et al. [28] studied the TAD fusion caused by deletion of CTCF sites in vivo in the mouse embryonic limb bud at the *Sox9-Kcnj2* locus. They used the promoter capture Hi-C data in the E12.5 mouse limb bud to show the structural changes after deleting major CTCF sites (mm9, GSE78109, GSE125294). In WT TAD structures, *Kcnj2* and *Sox9* are separated into two TADs. After deleting four consecutive CTCF sites within a 15 kb boundary region (C1 site mm9 chr11:111,384,818-111,385,832, C2-C4 site chr11:111,393,908–111,399,229), the TAD boundaries disappeared and the two TADs fused together. When all CTCF binding sites between *Kcnj2* and *Sox9* were deleted, they observed a more complete TAD fusion (Fig. 6A). These experiments revealed a TAD fusion caused by the deletion of major CTCF sites at the boundaries and within the TAD.

We then tested Epiphany’s ability to predict these structural changes after we perturbed the CTCF input track. Epiphany was trained on data from the human cell line GM12878 and used to make cross-species prediction in the mouse embryo. Epigenomic tracks were downloaded from ENCODE [29] and BioSamples [30] for mouse limb tissue aligned to the mm10 assembly (Table 4). In the WT prediction, Epiphany predicted a strong boundary separating *Kcnj2* and *Sox9* into two TADs. Upon deleting the C1–4 CTCF peaks (mm10 chr11:111,520,000–111,540,000) at the boundary and further masking all CTCF sites, Epiphany predicted behavior consistent with the ground truth experiments, where the two TADs gradually merged together (Fig. 6B, top). We further explored the relationship between the CTCF peaks and TAD formation using feature attribution methods (Fig. 6B, bottom). The SHAP values are calculated and averaged for the bins in the vertical highlighted dashed lines. We can see that CTCF peaks at the boundary contribute to TAD separation in unperturbed prediction, while with the masked CTCF track, the feature attribution scores focus on more distal regions at the boundaries of the fused TAD.

**Table 4:** Hi-C data source

Track	Cell type	Days	Accession Number	Source
DNaseI	Mouse limb buds	E11.5	ENCSR661HDP	ENCODE
CTCF	Mouse limb buds	E11.5	SAMD00019977	BioSamples
H3K27ac	Mouse limb buds	E12.5	ENCSR737QWV	ENCODE
H3K27me3	Mouse limb buds	E12.5	ENCSR229LTY	ENCODE
H3K4me3	Mouse limb buds	E12.5	ENCSR938MUD	ENCODE

We further used this example to compare the ability of the MSE-only and MSE+GAN models to make interpretable predictions of 3D structural changes under epigenomic perturbations, and the results are shown in Additional file 1: Fig. S14. In each panel, the top shows the contact map for WT, the middle shows the map after CTCF deletion, and the bottom shows the absolute difference between the two conditions. We can see that the MSE+GAN prediction identifies a clear TAD boundary disappearance after CTCF deletion, but the prediction from the MSE-only model less precisely shows the difference.

We also evaluated Epiphany on another prediction task of structural changes caused by genomic deletions. Wu et al. [31] experimented with CTCF perturbation at the *SOX17* locus in human ES cells. Figure 6C shows Capture Hi-C maps at the SOX17 locus before (top) and after (bottom) targeting the CTCF site. The deleted region (marked in dashed vertical lines) is very close to the *SOX17* distal regulatory element (DRE). The authors found that removing the CTCF peak at the boundary upstream of the *SOX17* locus leads to decreased interaction of *SOX17* and its DRE, and increased interaction with the upstream boundary near the *RGS20* locus. Figure 6D shows the Epiphany prediction at the *SOX17* region before (top) and after (bottom) CTCF deletion.

## Discussion

In this study, we developed Epiphany, a neural network model to predict the cell-type-specific Hi-C contact map for entire chromosomes up to a fixed genomic distance using commonly generated epigenomic tracks that are already available for diverse cell types and tissues. We showed that Epiphany accurately predicts cell-type-specific 3D genome architecture and shows robust performance for Hi-C different normalization procedures and at different resolutions. Epiphany was able to accurately predict cross-chromosome, cross-cell type and even cross-species 3D genomic structures. From feature ablation and attribution experiments, we showed that Epiphany could be used to interpret the contribution of specific epigenomic signals to local 3D structures. Through in silico perturbations of epigenomic tracks followed by contact map prediction with Epiphany, we were able to accurately predict the cell-type-specific impact of epigenetic alterations and structural variants on TAD organization in previously studied loci.

Epiphany’s ability to predict 3D structural changes given perturbations in epigenomic signals enables many interesting biological applications, such as assessing the 3D impact of germline or somatic structural variants or of inactivation of epigenomic elements. However, we would caution that Epiphany is only designed to predict the consequences of local epigenomic perturbations and has only been evaluated in this context. Massive global changes in histone marks—for example, through knockout or inhibition of epigenetic regulators like EZH2 or HDAC, or through loss-of-function or gain-of-function mutations in genes encoding epigenetic regulators—might in fact change the rules that govern the relationship between 1D and 3D chromatin organization. In future work, and as suitable datasets emerge, we hope to explore how the Epiphany model changes under global epigenetic perturbations.

Although we used five specific epigenomic tracks (DNase I, CTCF, H3K27ac, H3K27me3, H3K4me3) and Hi-C data in this study, Epiphany provides a general framework to link cell-type-specific epigenomic signals to 3D genomic structures. In the future, we plan to explore different combinations of the epigenomic input tracks to assess their biological and statistical relevance for prediction of 3D structure. In addition to using epigenomic information, we also tried to incorporate DNA information into the model. Previous models have used a one-hot encoding of long genomic DNA sequences ($\sim 1Mb$), incurring significant computational costs [12, 13]. We therefore tried an alternative strategy of extracting DNA representations from a pre-trained DNABERT model [32], a new method that adapts the state-of-the-art natural language processing model BERT [33] to the setting of genomic DNA. During the pre-training phase, DNA sequences were first truncated to 512 bp length sequences as the “sentences” and further divided into k-mers as the “words” of the vocabulary. The model learns the basic syntax and grammar of DNA sequences by self-supervised training to predict randomly masked k-mers within each sentence. After pre-training, each 512 bp sequence was represented by a 768-length numerical vector. However, since Epiphany covers a 3.4-Mb region as input during training, it was still extremely computationally intensive to directly incorporate the pre-trained representations from DNABERT. We therefore excluded the DNABERT component in order to keep the model relatively light-weight and concise, although we do not rule out its utility in the future.

Beyond these computational issues, a more conceptual modeling challenge is retaining the ability to generalize to new cell types while also incorporating DNA sequence information. In principle, training on genomic sequence may learn DNA sequence features that are specific to the training cell types and do not generalize to other cell types. Epiphany learns a general model for predicting the Hi-C contact map in a cell type of interest from cell-type-specific 1D epigenomic data, giving state-of-the-art prediction accuracy while allowing generalization across cell types and across species.

Many architectural changes and extensions could be investigated in future to build upon Epiphany. While we used a Bi-LSTM architecture to capture long-range dependencies in 1D epigenomic tracks that are useful for contact map prediction, other strategies such as dilated convolutional neural networks or transformers are possible. In the current Epiphany model, we provide the option of training with a GAN component and adversarial loss to help the model learn perceptually correct structures. In future work, we also hope to explore likelihood based generative models such as diffusion probabilistic models for Hi-C structural predictions.

## Conclusion

We present Epiphany, a neural network to predict cell-type-specific Hi-C contact maps from commonly generated epigenomic tracks. Epiphany employs 1D convolutional layers to learn the local representations from the input tracks, bidirectional long short-term memory (Bi-LSTM) layers to capture long term dependencies along the epigenome, as well as an optional generative adversarial network (GAN) architecture to encourage contact map realism. To improve the usability of predicted contact matrices, we trained and evaluated models using multiple normalization and matrix balancing techniques including KR, ICE, and HiC-DC+ *Z*-score and observed-over-expected count ratio. Epiphany is trained either with MSE alone or with a combination of MSE and adversarial (i.e., a GAN) loss to enhance its ability to produce realistic Hi-C contact maps for downstream analysis. Epiphany shows robust performance at different resolutions and among multiple normalization methods, and can generalize to held-out chromosomes within and across cell types and species. The predicted contact maps yield accurate TAD and significant interaction calls. Epiphany can be trained with different combinations of epigenomic signals, and study the contribution of specific epigenomic peaks to 3D architecture. At inference time, Epiphany can also be used to predict the structural changes caused by perturbations of epigenomic signals.

## Methods

### Data sources and pre-processing

#### Training and test sets

We used three human cell lines (GM12878, H1-hESC, K562) and one mouse cell line (mESC) for training and testing the model. All human data (Hi-C, ChIP-seq) were processed using the hg38 assembly and mouse data with mm10. For all experiments, chromosomes 3, 11, 17 were used as completely held-out data for testing.

#### Epigenomic data

All input epigenomic tracks including DNaseI, CTCF, H3K4me3, H3K27ac, H3K27me3 for genome assembly hg38 were downloaded from the ENCODE data portal [29]. Data were downloaded as bam files, and the replicates for each epigenomic track were merged using the pysam (https://github.com/pysam-developers/pysam) python module. We then converted merged bam files into bigWig files with deepTools [34] bamCoverage (binSize 10, RPGC normalization, other parameters as default). Genome-wide coverage bigWig tracks were later binned into 100-bp bins, and bin-level signals for the 5 epigenomic tracks were extracted as input data for the model.

#### Dissecting epigenomic data for input

For each 1Mb stripe that is orthogonal to the diagonal on the Hi-C map, we take in a 1.4-Mb window of epigenomic signal centered at this stripe as input (1 Mb covers the corresponding interaction bins of the stripe, with an additional 400 Kb on both sides of the input). Since we are predicting 200 consecutive stripes simultaneously, the total input region is 3.4Mb using an overlapping sliding window approach, where the window size is 1.4Mb for a single stripe and the step size is 10 kb. Therefore, for each 200 stripe prediction, we will have our input as a 3D tensor with size (5 tracks $\times$ 3.4 Mb $\times$ 200 windows).

#### Hi-C data

High quality and deeply sequenced Hi-C data as .hic format for all human and mouse cell lines were downloaded from 4DN data portal [1]. Data were binned at 5 kb and 10 kb resolution and normalized using multiple approaches. KR normalization was calculated by Juicer tools [17] and ICE normalization by the HiCExplorer package [18]. Observed-over-expected count ratio and *Z*-score normalizations were calculated by HiC-DC+ [16]. ICE normalization for 5 kb resolution was calculated using Cooler [35], and all additional matrix balancing steps followed the Akita pipeline [13]. For the observed-over-expected count ratios from HiC-DC+, raw counts for interaction bins are modeled using negative binomial regression to estimate a background model, giving an expected count value based on the genomic distance and other covariates associated with the anchor bins (GC content, mappability, effective size due to restriction enzyme site distribution). The observed-over-expected count ratio is then calculated using observed raw counts divided by the expected counts from the HiC-DC+ model.

#### Biological validation data

Capture Hi-C and corresponding CTCF tracks from Despang et al. [28] were downloaded from (GSE78109, GSE125294). Data were visualized using Coolbox [36].

### Model and training

#### CNN layers

The input epigenomic tracks were divided into overlapping windows, with a window length of $m = 14,000$ bins (1.4 Mb) and a stride of 1000 bins (100 kb). We refer to the windowed inputs as $X = \{x_1, ..., x_n\}$, where $x_i \in \mathbb {R}^{c \times m}$ corresponds to window *i*, *n* is the total number of windows, and *c* is the number of epigenomic tracks. A series of four convolution modules were used to featurize each window into a vector of dimension $d = 900$ (after flattening), where each convolution module consists of a convolutional layer with ReLU activation, max pooling, and dropout. We define $Z = \{z_1, ..., z_n\}$ as the flattened output of the final convolution module where $z_i \in \mathbb {R}^d$ is the representation for window $x_i$.

#### Bi-LSTM layers

The Bi-LSTM layers receive sequence $Z = \{z_1, ..., z_n\}$ as an input and generate a new sequence $\tilde{Z} = \{\tilde{z}_1, ..., \tilde{z}_n\}$, where $\tilde{z}_i \in \mathbb {R}^{2d}$. To produce the final output, every element of $\tilde{Z}$ is passed through a fully connected layer yielding the output sequence $\hat{Y} = \{\hat{y}_1, ..., \hat{y}_n\}$. Each $\hat{y}_i \in \mathbb {R}^{d'}$ is a vector of dimension $d' = 100$ (or $d' = 200$ if predicting 5 kb resolution Hi-C) and corresponds to a *zig-zag* stripe in a Hi-C matrix, similar to DeepC (shown in Fig. 1). Epiphany uses multiple Bi-LSTM layers, with skip connections between successive layers.

#### Adversarial loss

Generative adversarial networks (GAN) consist of two networks, a generator $\mathcal {G}$ with parameters $\theta ^{\mathcal {G}}$ and a discriminator $\mathcal {D}$ with parameters $\theta ^{\mathcal {D}}$, that are adversarially trained in a zero-sum game [21, 37]. During training, the generator learns to fool the discriminator by synthesizing realistic samples from a given input, while the discriminator learns to distinguish real samples from synthetic samples. To train Epiphany in the MSE+GAN mode, we employed a convex combination of pixel-wise MSE and adversarial loss. Given a dataset *D* and a trade-off parameter $\lambda$, the MSE+GAN version of Epiphany solves the following optimization problem during training:

2 $$\begin{aligned} \min _{\theta ^{\mathcal {G}}} \max _{\theta ^{\mathcal {D}}} \lambda \mathcal {L}_{adv}(\theta ^{\mathcal {G}}, \theta ^{\mathcal {D}}) + (1 - \lambda )\mathcal {L}_{MSE}(\theta ^{\mathcal {G}}) \end{aligned}$$

3 $$\begin{aligned} \mathcal {L}_{adv}(\theta ^{\mathcal {G}}, \theta ^{\mathcal {D}}) = \mathbb {E}_{(X,Y) \sim D} \left[ \log (\mathcal {D}(Y)) + \log (1 - \mathcal {D}(\mathcal {G}(X))) \right] \end{aligned}$$

4 $$\begin{aligned} \mathcal {L}_{MSE}(\theta ^{\mathcal {G}}) = \mathbb {E}_{(X,Y) \sim D} \left[ \sum _{i \in [n]} \sum _{j \in [d']} \left( Y_{ij} - [\mathcal {G}(X)]_{ij} \right) ^2 \right] , \end{aligned}$$

where *X* corresponds to epigenomic tracks and *Y* the corresponding Hi-C matrix.

In our framework, $\mathcal {G}$ is the CNN-LSTM architecture described in the previous sections while $\mathcal {D}$ is a simple four layer 2D CNN. Note that in practice, many tricks and heuristics are used in order to speed up convergence when training GANs, as described below.

#### Training

In Algorithm 1, we show the specific procedure used to approximately solve the optimization problem described above. Note that rather than setting $\mathcal {L_D}$ to $-\mathcal {L_G}$, we employ the target flipping heuristic outlined in [21] (Section 3.2.3) for faster convergence. The parameter updates (lines 6 and 9) are computed via the Adam optimizer [38]. During training, we randomly sample a Hi-C region consisting of 200 consecutive bins as the target, and use a 3.4Mb subsequence of the epigenetic tracks as an input for each gradient computation. We determine when to conclude training based on when $\mathcal {L}_G$ ceases to decrease.

**Figure:**
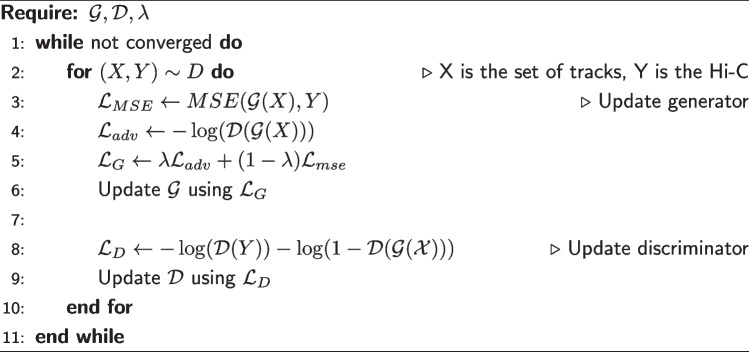
**Algorithm 1** Epiphany Training

### Performance evaluation and application

#### Model performance

We evaluated the model performance using Pearson and Spearman correlation of the predicted contact map vs. ground truth, computed as a function of genomic distance from the diagonal. Predicted contact maps were saved as .hic files for downstream analysis. We visualized Hi-C matrices and epigenomic tracks using CoolBox [36]. The insulation score was calculated using the TAD-separation score from HiCExplorer [39]. Then a correlation of these scores between ground truth vs. predicted contact maps was calculated. For each 2 Mb length submatrix (200 bin matrix), we calculated MSE loss and insulation score correlation between the predicted and true maps.

#### TAD boundaries and significant interactions

We identified TAD structures and significant interactions in the predicted contact maps vs. ground truth. TAD structures were identified using TopDom [23], with various window sizes of 10, 20, 30, 40, 50. Since the binary TAD boundaries would be less robust towards hyper-parameter selection and small value perturbations on the contact maps, we used insulation score for the comparisons. In this evaluation, we first ran TopDom on all ground truth contact maps with different normalization methods, and used KR normalization as the gold standard, to compare the agreement between these normalization approaches (e.g., ICE vs. KR, *Z*-score vs. KR). We then compared TopDom results called from predicted contact map vs. the ground truth with their corresponding normalization approach (e.g., predicted *Z*-score vs. ground truth *Z*-score), to evaluate Epiphany’s ability to predict key structures. These experiments were all run on test chromosomes 3, 11, 17.

HiC-DC+ [16] used interaction bin counts to fit a negative binomial regression with genomic distance, GC content, mappability and effective bin size based on restriction enzyme sites, providing an estimated expected read count for each interaction bins. *Z*-scores and observed-over-expected count ratios are then computed to evaluate the significance of the observed counts. We defined significant interactions as ground truth *Z*-score greater than or equal to 2. For test chromosomes 3, 11, 17 with *Z*-score and observed-over-expected count ratio normalization, we called significant interactions with various cut-off thresholds ranging from 0.5 to 3.5 and plotted the ROC curve.

#### Comparison with Akita

We followed provided tutorials and extracted the pre-trained Akita model from (Akita repository). Hi-C contact maps were first balanced using ICE normalization, followed by additional steps including adaptive coarse-grain, distance adjustment, rescaling and 2D Gaussian filter suggested by Akita. Test matrices were extracted from Akita held-out test regions that overlapped with Epiphany’s test chromosomes (42 regions in total). For calculating the Pearson correlation between the predicted contact map vs. ground truth, we average-pooled Akita matrices from 2048 bp into 4096 bp, in order to keep relative consistency with our 5 kb resolution. For extracting cell-type-specific predictions, we extracted the multi-task output from Akita for H1-hESC and GM12878.

#### Prediction of cell-type-specific structures

In these experiments, Epiphany was trained on the training chromosomes in GM12878, and tested on test chromosomes chr 3, 11, 17 on H1-hESC and K562. Therefore, the predictions were cross-chromosome and cross-cell-type. Feature attributions were calculated using Captum [40], with saliency score to show the gradient attribution on input regions and SHAP values to calculate the contribution of specific epigenomic peaks for predicting 3D structure. The baseline was set to zero when calculating SHAP values.

#### Feature ablation models

Feature ablation experiments were performed by re-training the model with one or several input epigenomic tracks completely masked as zero. We tested three ablation models: CTCF masked; DNaseI masked; only CTCF and H3K27ac not masked. In addition, we also re-trained Epiphany with an additional SMC3 ChIP-seq track to include cohesin occupancy information. Whole chromosome predictions were generated with trained models and compared to ground truth using Pearson and Spearman correlations as a function of genomic distance. Feature attributions were calculated as described above.

#### Biological application on mouse data

Epigenomic tracks for mouse limb bud tissue using genome assembly mm10 were downloaded from the ENCODE portal. In the CTCF deletion experiments, CTCF peaks were masked with the average value for the entire CTCF track (masked with the background). Epiphany was trained on the human cell line GM12878 and tested on mouse limb bud data (E11.5 for DNaseI and CTCF tracks, and E12.5 for H3K27ac, H3K27me3 and H3K4me3). Data were visualized using CoolBox [36].

### Supplementary information

**Additional file 1:** Supplementary information. **Fig. S1** MSE and GAN score for Hi-C matrix evaluations. **Fig. S2** Binarized predictions show incorrect structure for MSE-only model. **Fig. S3** Evaluating predictions with well-trained discriminator. **Fig. S4** Ground truth, Epiphany predictions with epigenomic tracks. **Fig. S5** TAD calling evaluation via Pearson correlation of insulation scores. **Fig. S6** Performance of MSE-onlyand MSE+GANmodels using observed-over-expected count ratio target for detection of significant interactions. **Fig. S7** Comparison between Akita and Epiphany on GM12878 and Hi-hESC. **Fig. S8** Genome-wide evaluation of cell-type specific predictions on K562. **Fig. S9** Genome-wide evaluation of cell-type-specific predictions in heart left ventricle. **Fig. S10** Cell-type-specific predictions in K562 compared with Akita. **Fig. S11** Cell-type-specific prediction of Epiphany. **Fig. S12** Comparison of K562 and HCT116 on conserved and non-conserved regions. **Fig. S13** Visual comparison of DNaseI + CTCF only prediction. **Fig. S14** Predicting 3D structural changes after CTCF deletion with the MSE+GAN and MSE-only models.

**Additional file 2.** Pearson correlation and ***p***-values for comparing Akita and Epiphany model on GM12878.

**Additional file 3.** Conserved regions between the two cell types. Regions between K562 and HCT116 are compared by hicDifferentialTAD function from HiCExplorer. Regions failed to reject H0 ‛regions are equal′ with ***p***-value greater than 0.01 are identified as conserved regions between the two cell types.

**Additional file 4.** Non-conserved regions between the two cell types. Regions with *p*-value less than 0.01 are identified as non-conserved regions.

**Additional file 5.** Pearson correlation of insulation scores called by TopDom between predictions using MSE-onlyand GAN.

**Additional file 6.** Review history.

## Data Availability

Datasets in this study are all publicly available. Code can be found on github under the MIT license [41]. Pretrained weights for Epiphany model (5kb and 10kb resolution), as well as sample input and target datasets can be found at Zenodo [42]. Hi-C data are available from the 4DN data portal. All human data are with hg38 assembly, and mouse with mm10. Accession numbers are in Table 2. Epigenomic tracks for human are available from ENCODE. Accession numbers are in Table 3. Cohesin for GM12878 is available at ENCSR000DZP. In additional experiments, capture Hi-C data and CTCF tracks for mES E12.5 with mm9 assembly for biological validation from Despang et al. [28] are publicly available at GSE78109, GSE125294. Epigenomic tracks for mouse limb validation are available at ENCODE [29] and BioSamples [30]. Accession numbers are in Table 4. Capture Hi-C data for H1-hESC with hg19 assembly for biological validation from Wu et al. [31] are publicly available at GSE127196.
