# Author Correction: Epiphany: predicting Hi-C contact maps from 1D epigenomic signals

**DOI:** 10.1186/s13059-024-03281-z

**Published:** 2024-05-23

**Authors:** Rui Yang, Arnav Das, Vianne R. Gao, Alireza Karbalayghareh, William S. Noble, Jeffrey A. Bilmes, Christina S. Leslie

**Affiliations:** 1 Memorial Sloan Kettering Cancer Center, New York, USA; 2 University of Washington, Seattle, USA

**Correction: Genome Biol 24, 134 (2023)**

**https://doi.org/10.1186/s13059-023-02934-9**

Following publication of the original article [1], the authors identified an error in the given name of author Jeffrey A. Bilmes.

The incorrect author name is: Jeffery A. Bilmes

The correct author name is: Jeffrey A. Bilmes

The author group has been updated above and the original article [1] has been corrected.
