# Supplementary material for: Epiphany: predicting Hi-C contact maps from 1D epigenomic signals
Source: Genome Biol. 2023 Jun 6;24:134. doi: 10.1186/s13059-023-02934-9 (PMC10242996; doi:10.1186/s13059-023-02934-9)
Supplement: Supplementary file 1 — Additional file 1: Supplementary information. Fig. S1 MSE and GAN score for Hi-C matrix evaluations. Fig. S2 Binarized predictions show incorrect structure for MSE-only model. Fig. S3 Evaluating predictions with well-trained discriminator. Fig. S4 Ground truth, Epiphany predictions with epigenomic tracks. Fig. S5 TAD calling evaluation via Pearson correlation of insulation scores. Fig. S6 Performance of MSE-onlyand MSE+GANmodels using observed-over-expected count ratio target for detection of significant interactions. Fig. S7 Comparison between Akita and Epiphany on GM12878 and Hi-hESC. Fig. S8 Genome-wide evaluation of cell-type specific predictions on K562. Fig. S9 Genome-wide evaluation of cell-type-specific predictions in heart left ventricle. Fig. S10 Cell-type-specific predictions in K562 compared with Akita. Fig. S11 Cell-type-specific prediction of Epiphany. Fig. S12 Comparison of K562 and HCT116 on conserved and non-conserved regions. Fig. S13 Visual comparison of DNaseI + CTCF only prediction. Fig. S14 Predicting 3D structural changes after CTCF deletion with the MSE+GAN and MSE-only models. [file 13059_2023_2934_MOESM1_ESM.pdf]

Supplementary Information

Model Architecture Details

In the following tables, we describe in detail Epiphany's architecture. In each table, n is the total number of Hi-C stripes we seek to predict.

Operation	Number of Filters	Filter Size	Stride	Activation	Output Shape
Input	-	-	-	-	n x 5 x 14000
Convolution	70	5 x 17	1	ReLU	n x 70 x 13984
Max Pool	1	1 x 4	1	-	n x 70 x 3496
Dropout (p = .1)	-	-	-	-	n x 70 x 3496
Convolution	90	70 x 7	1	ReLU	n x 90 x 3490
Max Pool	1	1 x 4	1	-	n x 90 x 872
Dropout (p = .1)	-	-	-	-	n x 90 x 872
Convolution	70	90 x 5	1	ReLU	n x 70 x 868
Max Pool	1	1 x 4	1	-	n x 70 x 217
Dropout (p = .1)	-	-	-	-	n x 70 x 217
Convolution	20	70 x 5	1	ReLU	n x 20 x 213
Adaptive Max Pool	1	-	1	-	n x 20 x 45
Dropout (p = .1)	-	-	-	-	n x 20 x 45
Flatten	-	-	-	-	n x 900

Table 1: Parameterization for 1D CNN for 5kb and 10kb Hi-C prediction

Operation	Hidden Layer Size	Activation	Output Shape
Bi-LSTM	1200	ReLU	n x 2400
Bi-LSTM	1200	ReLU	n x 2400
Bi-LSTM	2400	ReLU	n x 2400
Dense	-	ReLU	n x 900
Dense	-	None	n x 100

Table 2: Parameterization for Bi-LSTM for 10kb Hi-C prediction

Operation	Hidden Layer Size	Activation	Output Shape
Bi-LSTM	2400	ReLU	n x 4800
Bi-LSTM	2400	ReLU	n x 4800
Dense	-	ReLU	n x 1200
Dense	-	None	n x 200

Table 3: Parameterization for Bi-LSTM for 5kb Hi-C prediction

Supplementary Figures

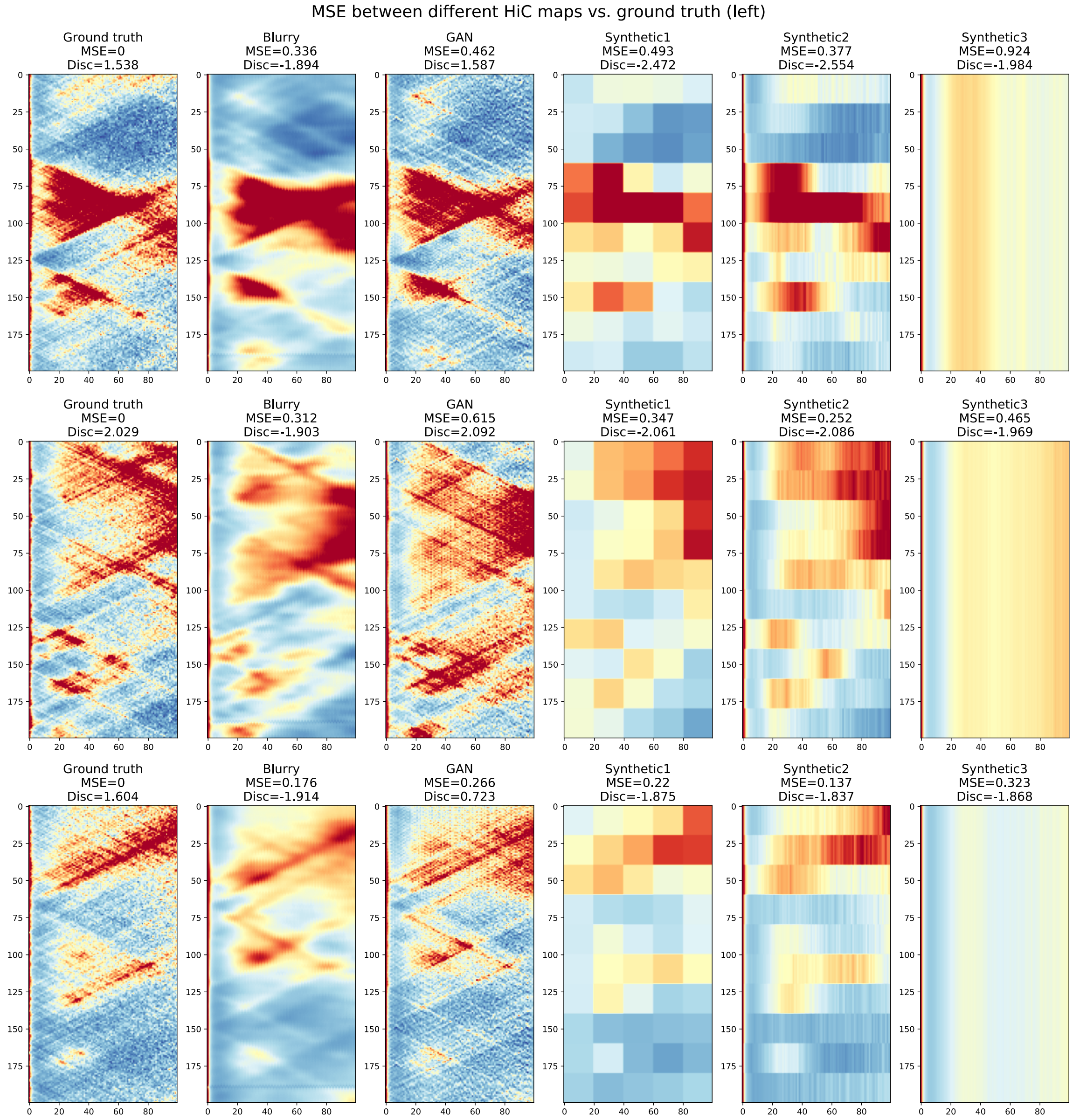

Supplementary Figure S1: MSE and GAN score for Hi-C matrix evaluations. Three random example on test chromosome 3 are shown. Each Hi-C map is rotated: the y-axis on the left shows the genomic coordinates, and the x-axis shows the distance from the diagonal. From left to right we plot: the ground truth Hi-C map, the prediction from MSE model, the prediction from GAN model, synthetic Hi-C map 1 (local average of the Hi-C map), synthetic Hi-C map 2 (local average for each genomic distance on the Hi-C map), synthetic Hi-C map 3 (averaged value at each genomic distance). The MSE value compared with ground truth, and the discriminator score calculated from the well-trained discriminator are shown in the subtitles.

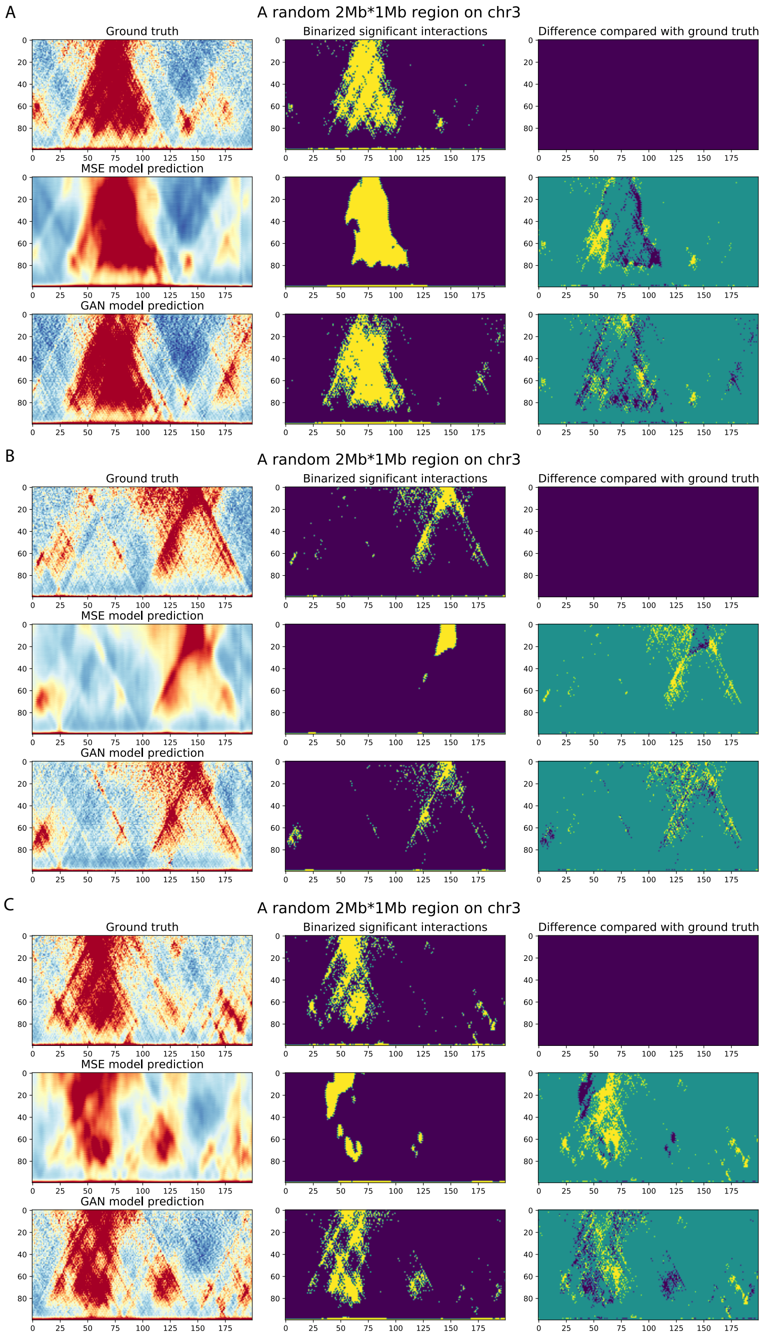

Supplementary Figure S2: Binarized predictions show incorrect structure for MSE-only model. In each example, a random region on test chromosome 3 of GM12878 (size: 2Mb along the diagonal, 1Mb from the diagonal) is shown. The left column shows the Hi-C map of ground truth (top), prediction from MSE-only trained model (middle), and prediction from the MSE+GAN model (bottom), using observed-over-expected count ratio as target values. The middle column shows the significant interactions called from each map, where interactions >= 2 are marked as 1 ("significant"), and interaction < 2 are marked as 0 ("not significant"). The right column shows the absolute difference of the significance plot (green=0, no difference; yellow = 1, false negatives, purple = -1, false positives). **(A)** 1496 bins were falsely predicted in the MSE-only blurry prediction, and 1815 bins falsely predicted in the MSE+GAN model prediction. **(B)** 1003 falsely predicted bins for the MSE-only model and 1249 for the MSE+GAN model. In this case, the MSE-only model detects a blob at the apex of the TAD but misses the edge structure. **(C)** 1962 bins vs. 2007 bins falsely predicted in MSE-only and MSE+GAN model.

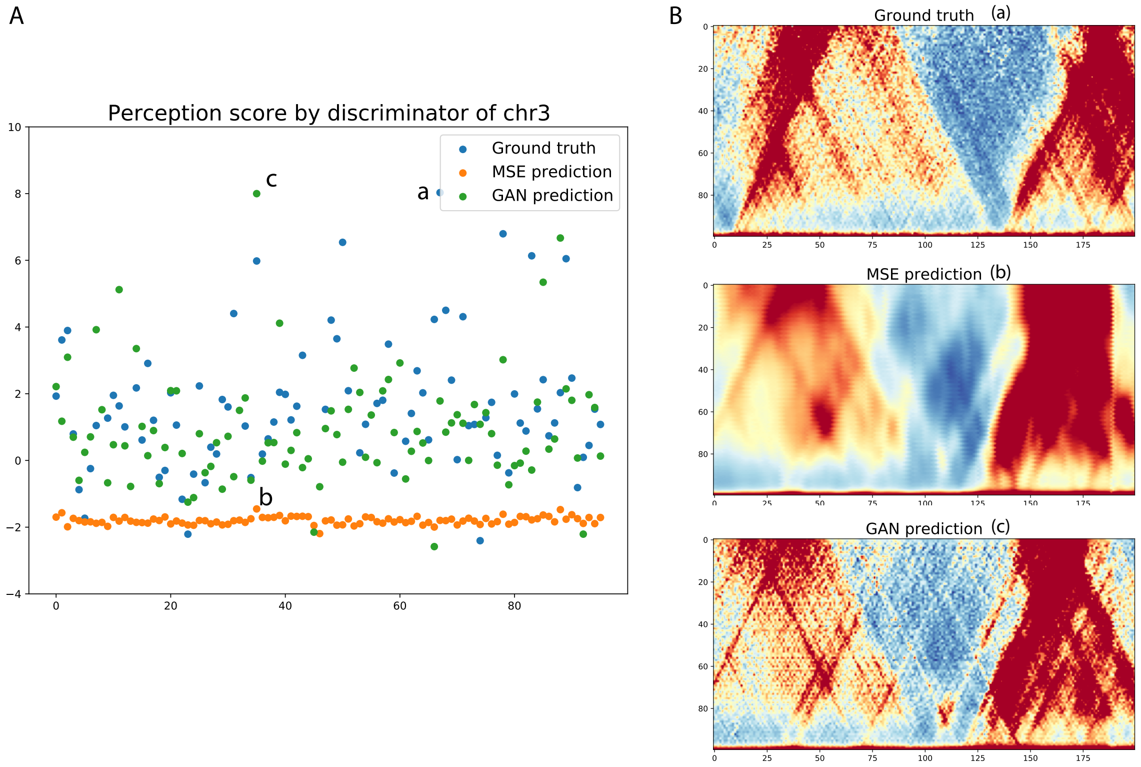

Supplementary Figure S3: Evaluating predictions with well-trained discriminator. (A) Perceptual score for all sub-regions (200×100, 2Mb along the diagonal, 1M from the diagonal) predicted on chr3. X-axis shows the genomic location of each 2Mb×1Mb sub-region along the diagonal, and y-axis shows the perceptual score. Blue dots show the perceptual score for ground truth Hi-C, green dots for the GAN prediction, and orange for the MSE-only prediction. All contact maps predicted by the MSE-only model obtain very low perceptual scores. **(B)** Example regions with the highest perceptual score in ground truth (a), MSE-only prediction (b), and MSE+GAN model prediction (c). Note that the ground truth example (a) corresponds to a different genomic location than the two predictions (b) and (c).

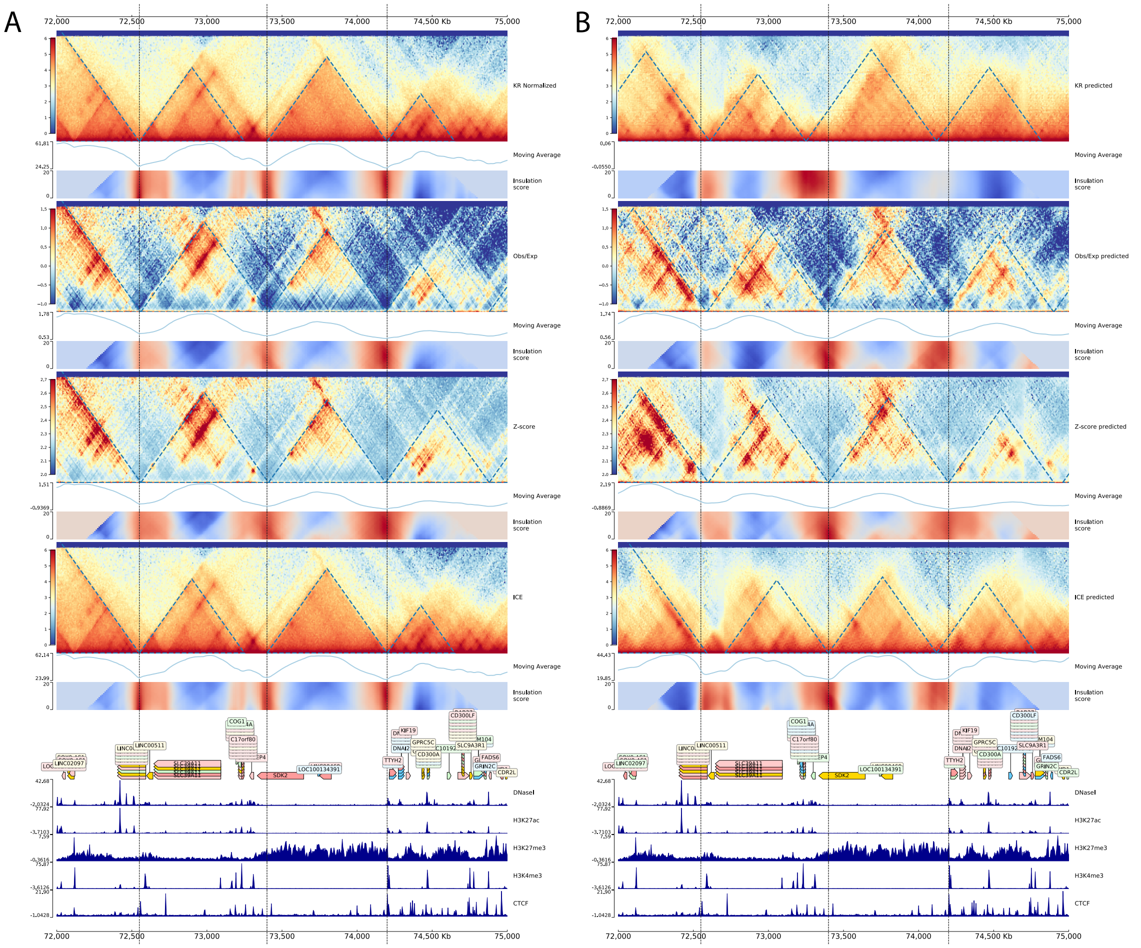

Supplementary Figure S4: Ground truth, Epiphany predictions with epigenomic tracks. Pearson correlation of insulation score on the three test chromosomes (chr3, 11, 17) of MSE only model vs. ground truth (blue), and MSE+GAN model vs. ground truth (red).

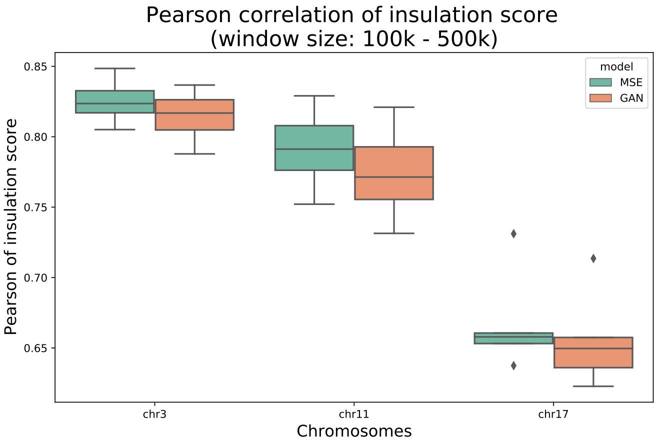

Supplementary Figure S5: TAD calling evaluation via Pearson correlation of insulation scores. Pearson correlation of insulation score on the three test chromosomes (chr3, 11, 17) of MSE only model vs. ground truth (blue), and MSE+GAN model vs. ground truth (red). Pearson correlation table is attached in Additional file 5.

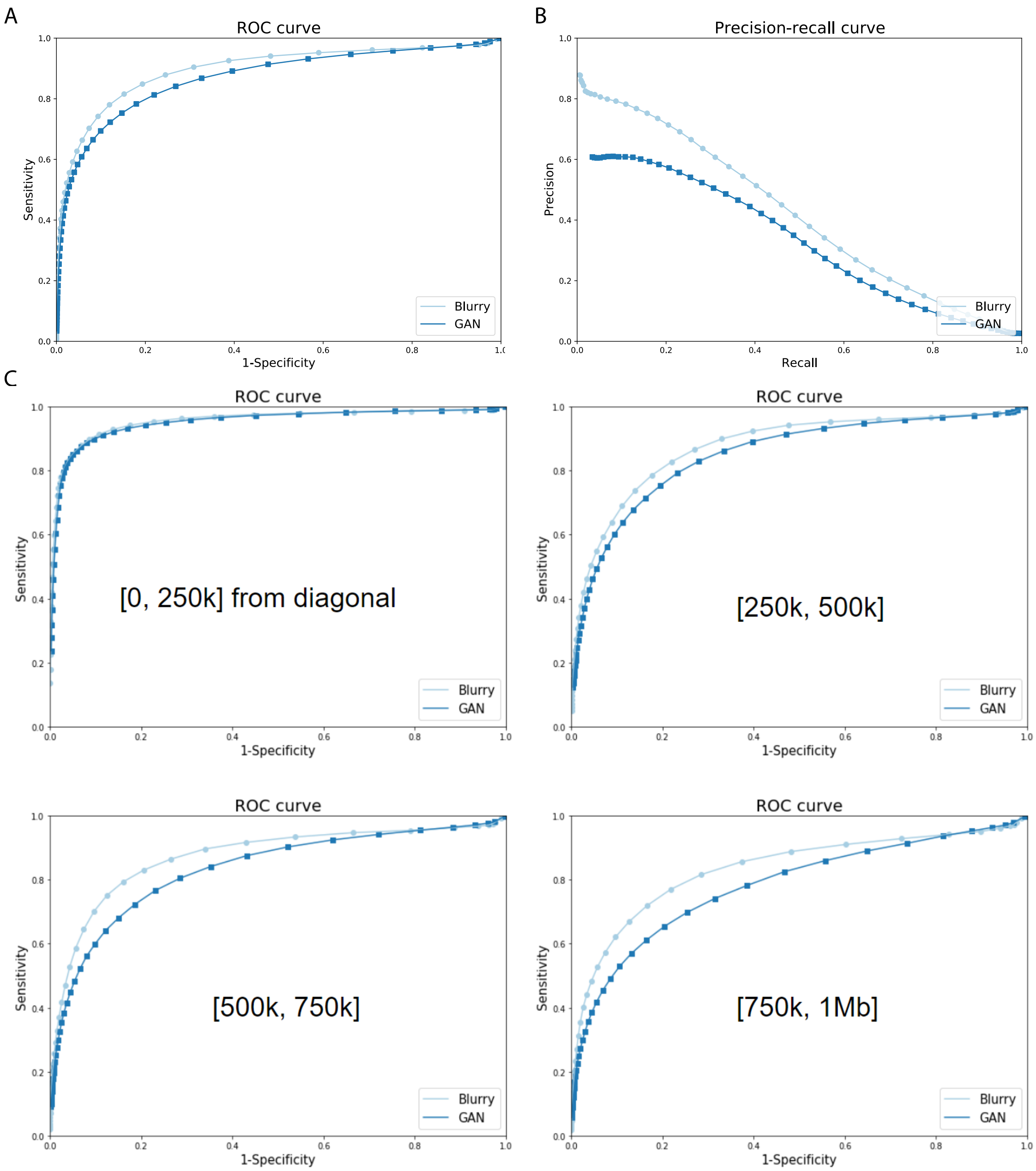

Supplementary Figure S6: Performance of MSE-only ("blurry") and MSE+GAN ("GAN") models using observed-over-expected count ratio target for detection of significant interactions. (A) ROC curve for the two models for detection of significant interactions. True positives defined by ground truth HiC-DC+ z-score of 3 or greater. **(B)** Precision-recall curve of the two models. **(C)** Genomic distance-stratified ROC curve.

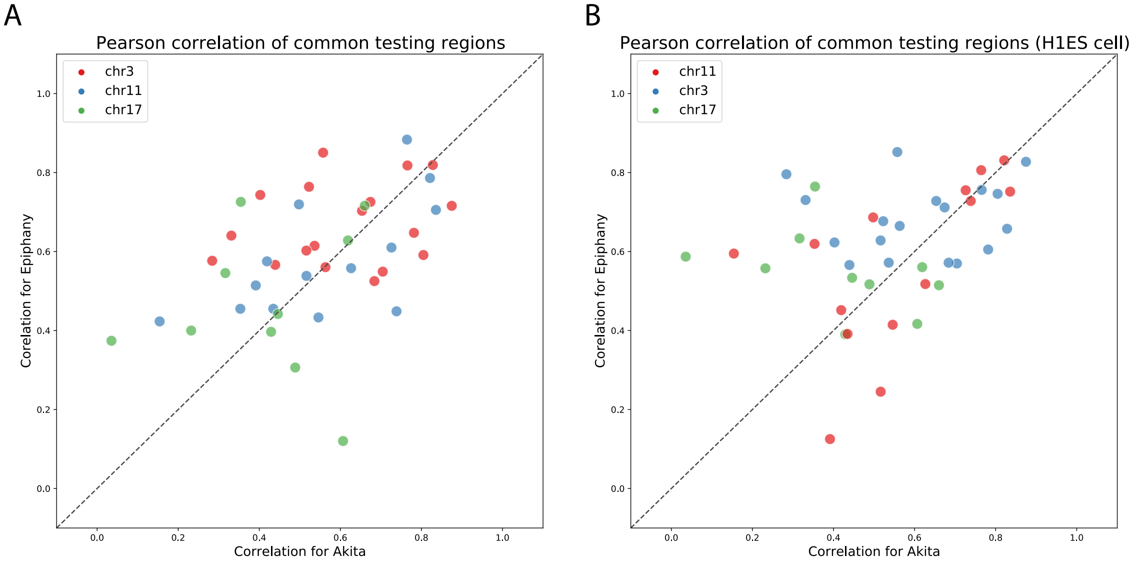

Supplementary Figure S7: Comparison between Akita and Epiphany on GM12878 and H1-hESC. **(A)** Model performance comparison between Epiphany and Akita on 42 common regions between Akita held-out test regions and our test chromosomes (chr3, 11, 17) in GM12878. The x-axis shows the Pearson correlation of Akita prediction vs. ground truth, and the y-axis shows the correlation of Epiphany. Epiphany was re-trained using data with the same normalization steps of Akita at 5 kb resolution, and Akita predictions were average-pooled into 4096 bp resolution for better comparison. Dots are colored by chromosomes. **(B)** Model performance comparison on H1-h1ESC.

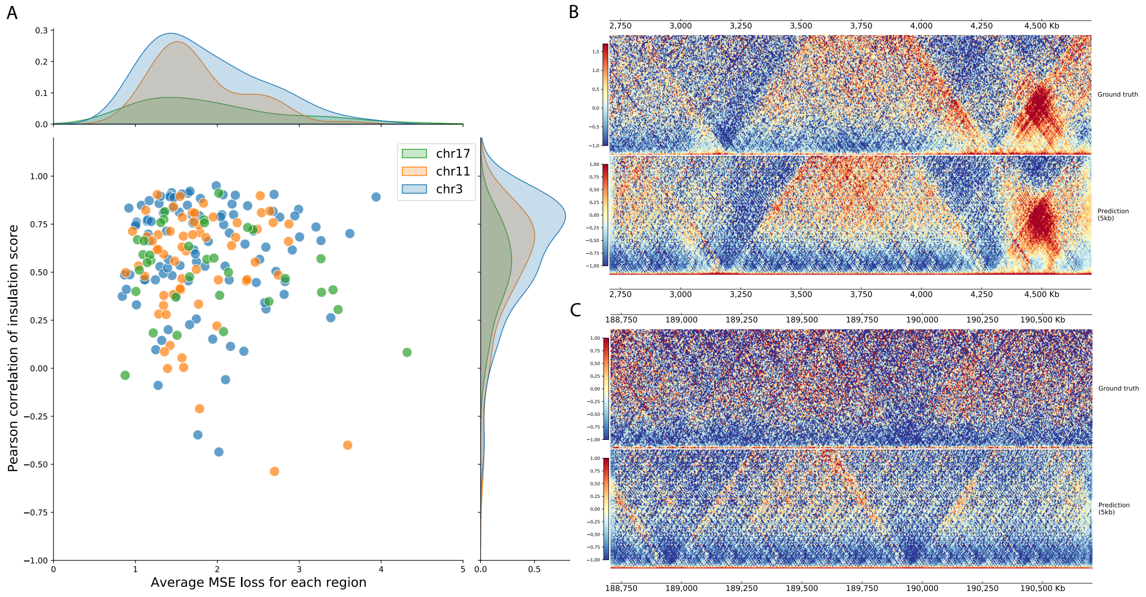

Supplementary Figure S8: Genome-wide evaluation of cell-type specific prediction on K562. Epiphany is trained on GM12878 training chromosomes (all chromosomes except for 3, 11, 17), and tested on K562 (cross-cell type, cross-chromosomal prediction). **(A)** An overview of Epiphany's performance on K562. Each dot corresponds to a 2Mb along the diagonal by 1Mb from the diagonal region, colored by chromosome. The x-axis shows the average MSE loss between Epiphany's prediction vs. ground truth, and the y-axis shows the Pearson correlation of the insulation score in this region. The majority of the regions have a Pearson correlation higher than 0.5. **(B)** A well predicted region. **(C)** A poorly predicted region.

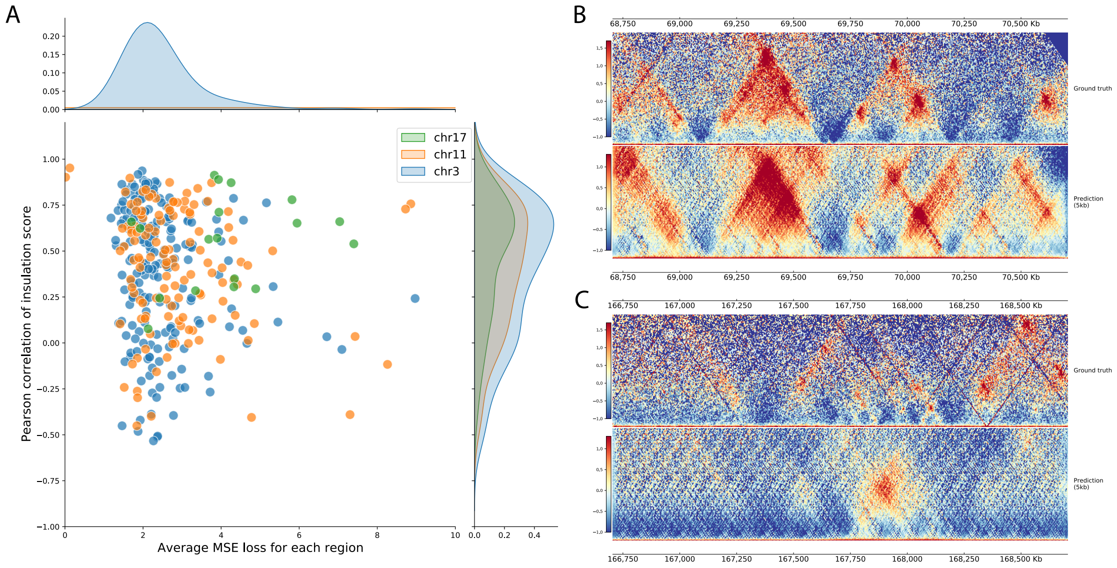

Supplementary Figure S9: Genome-wide evaluation of cell-type-specific predictions in heart left ventricle. Epiphany is trained on GM12878 training chromosomes (all chromosomes except for 3, 11, 17) and tested on heart left ventricle (cross-cell type, cross-chromosomal prediction). **(A)** An overview of Epiphany's performance on heart left ventricle. Each dot corresponds to a 2Mb along the diagonal by 1Mb from the diagonal region, colored by chromosome. The x-axis shows the average MSE loss between Epiphany's prediction vs. ground truth, and the y-axis shows the Pearson correlation of the insulation score in this region. The majority of the regions have a Pearson correlation higher than 0.5. **(B)** A well predicted region. **(C)** A poorly predicted region.

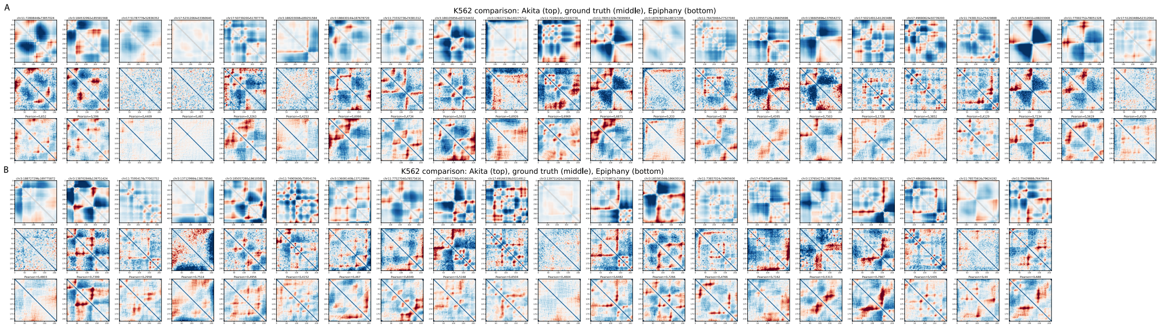

Supplementary Figure S10: Cell-type-specific predictions in K562 compared with Akita. Epiphany is trained on GM12878 training chromosomes (all chromosomes except for 3, 11, 17) and tested on K562 (cross-cell type, cross-chromosomal prediction). Akita prediction is the averaged prediction of five pre-trained cell types including GM12878, H1-hESC, HFF, HCT116, IMR90. **(A)** Conserved regions. Top row: averaged Akita prediction of 5 cell types (GM12878, hH1-hESC, IMR90, HCT116, HFF) on test regions. Middle row: ground truth contact map on K562. Bottom row: Epiphany prediction on K562. Pearson correlations between Epiphany prediction and ground truth are on the top. **(B)** Non-conserved regions. Rows as in **(A)**.

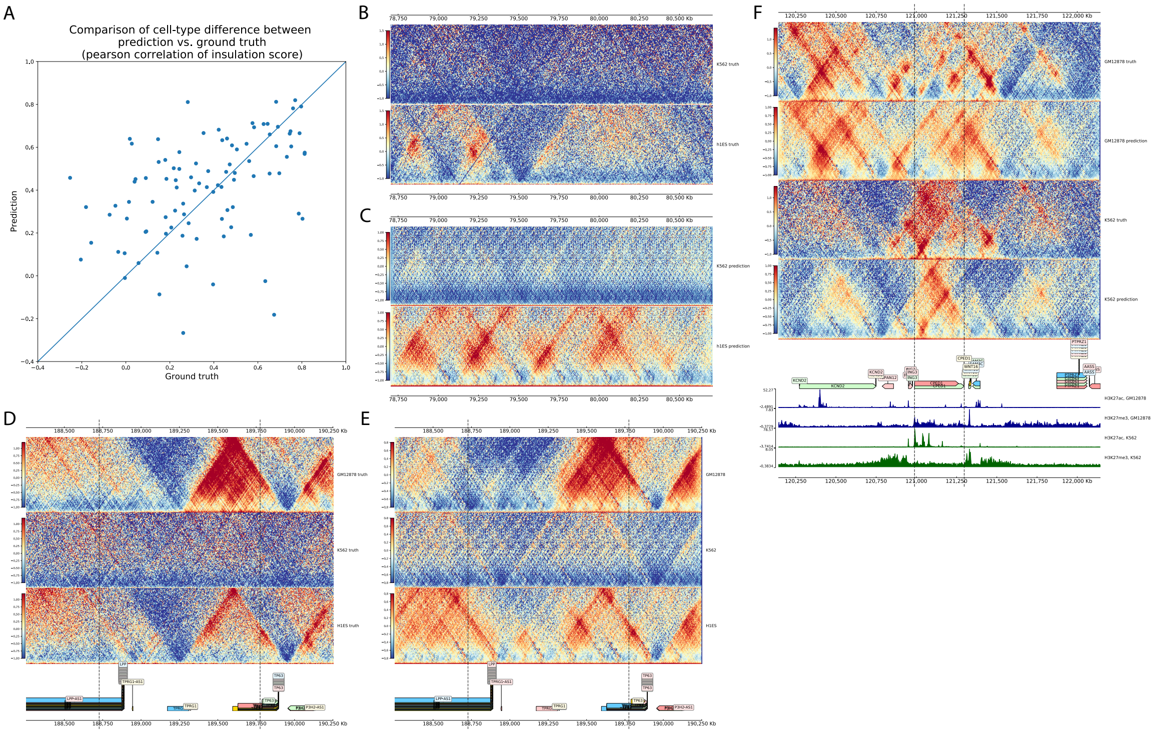

Supplementary Figure S11: Cell-type specific prediction of Epiphany. (A) Pearson correlation of the insulation scores between H1-hESC and K562 cells in ground truth (x-axis) and Epiphany predictions (y-axis). **(B)** Ground truth of a cell-type specific region (chr3:78,705,000-80,705,000) between H1-hESC (top) and K562 (bottom). **(C)** Epiphany prediction of the cell-type specific region between the two cell types. **(D)** Ground truth of a cell-type specific region (chr3:188,250,000-190,250,000) in GM12878 (top), K562 (middle) and H1-hESC (bottom). **(E)** Epiphany's cell-type specific prediction of the region (chr3:188,250,000-190,250,000). **(F)** Cell-type specific prediction around gene CPED1 locus: (top to bottom) GM12878 ground truth structure, GM12878 contact map predicted by Epiphany; K562 ground truth, K562 contact map predicted by Epiphany; gene annotations; and epigenomic tracks (H3K27ac, H3K27me3) for the two cell types.

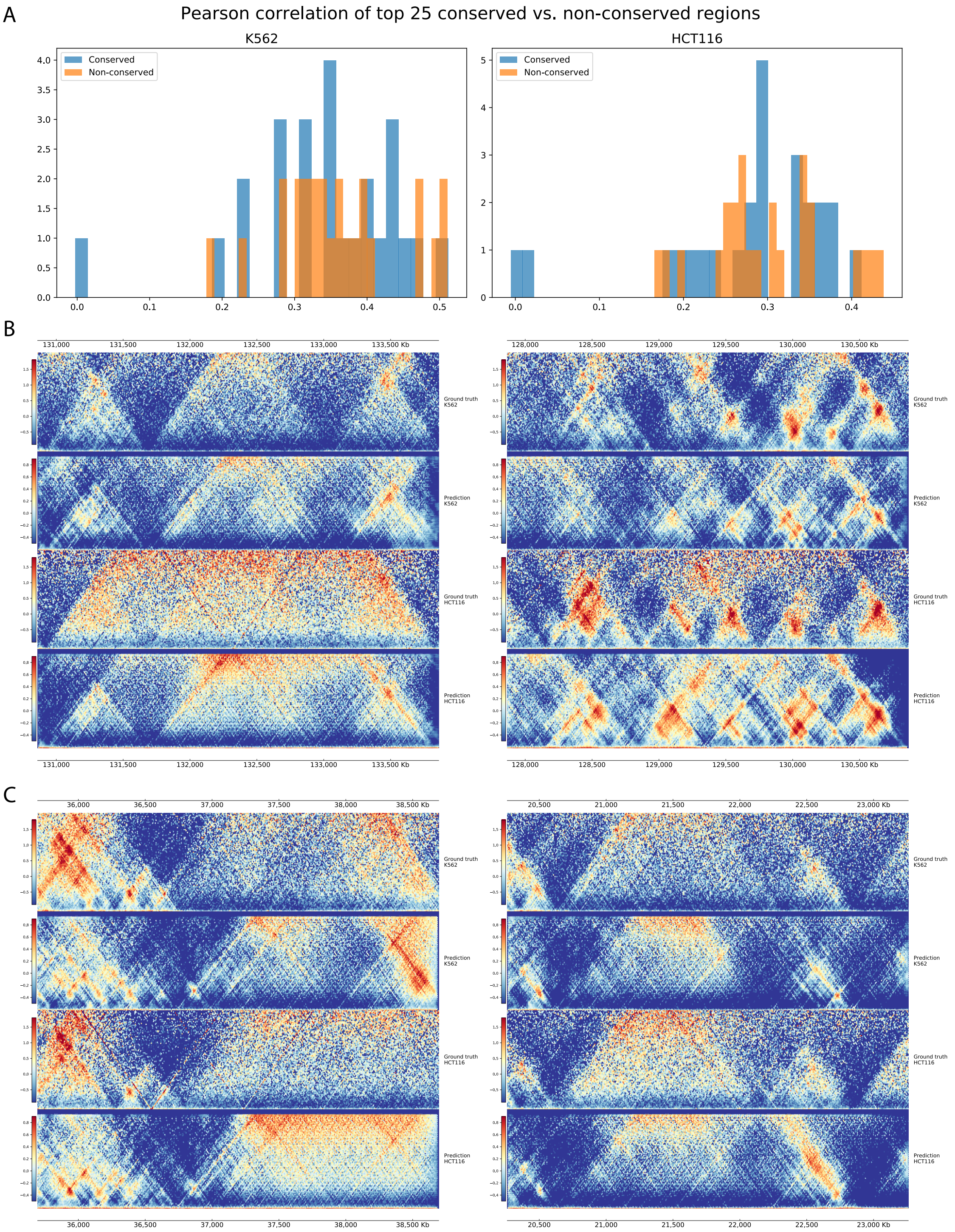

Supplementary Figure S12: Comparison of K562 and HCT116 on conserved and non-conserved regions. Epiphany is trained on GM12878 training chromosomes (all chromosomes except for 3, 11, 17), and tested on chromosome 11 for K562 and HCT116. **(A)** Pearson correlation between prediction vs. ground truth for top 25 conserved regions (blue) and non-conserved regions (orange) for K562 and HCT116. Conserved and non-conserved regions between the two cell types are identified by hicExplorer. **(B)** Visual examples of ground truth and prediction of top non-conserved regions. Top to bottom: K562 ground truth, K562 prediction, HCT116 ground truth, HCT116 prediction. **(C)** Visual examples of ground truth and prediction of top conserved regions between the two cell types.

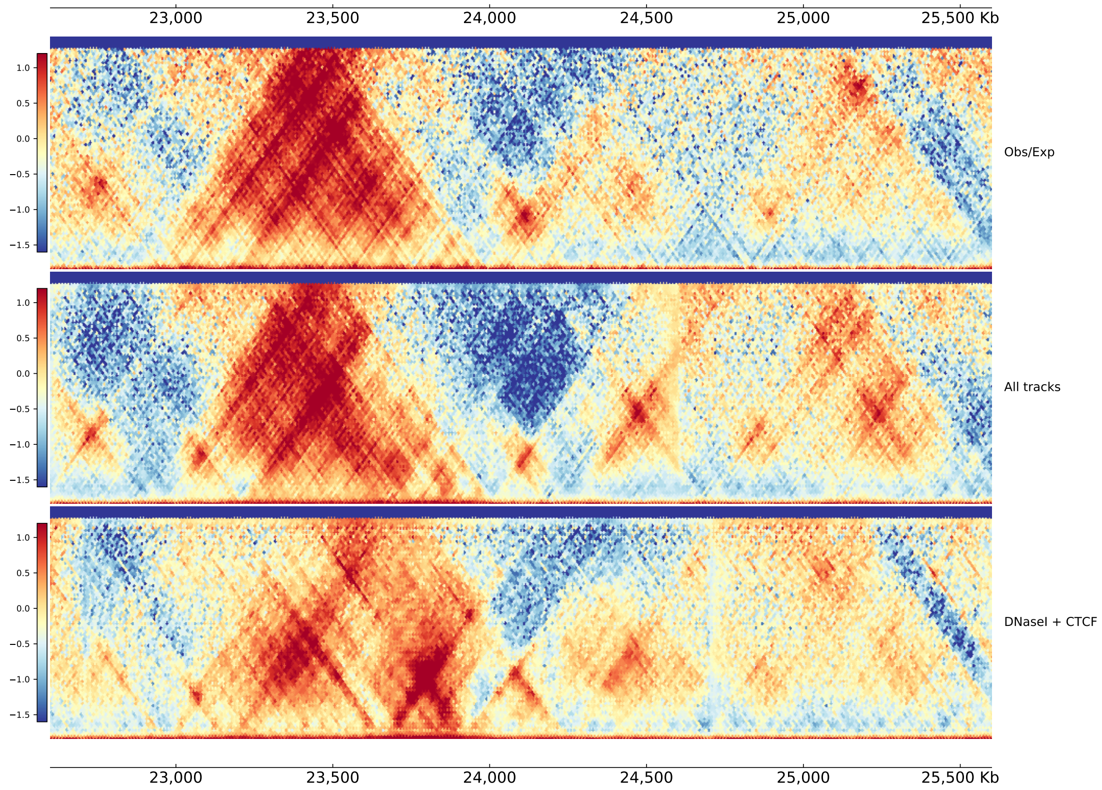

Supplementary Figure S13: Visual comparison of DNaseI + CTCF only prediction. A random region (chr3:123,675,000-126,675,000) in test chromosome 3 for ground truth (top), Epiphany prediction using all five tracks: DNaseI, H3K27ac, H3K27me3, H3K4me4, CTCF (middle), and Epiphany prediction using only two tracks: DNaseI, CTCF (bottom).

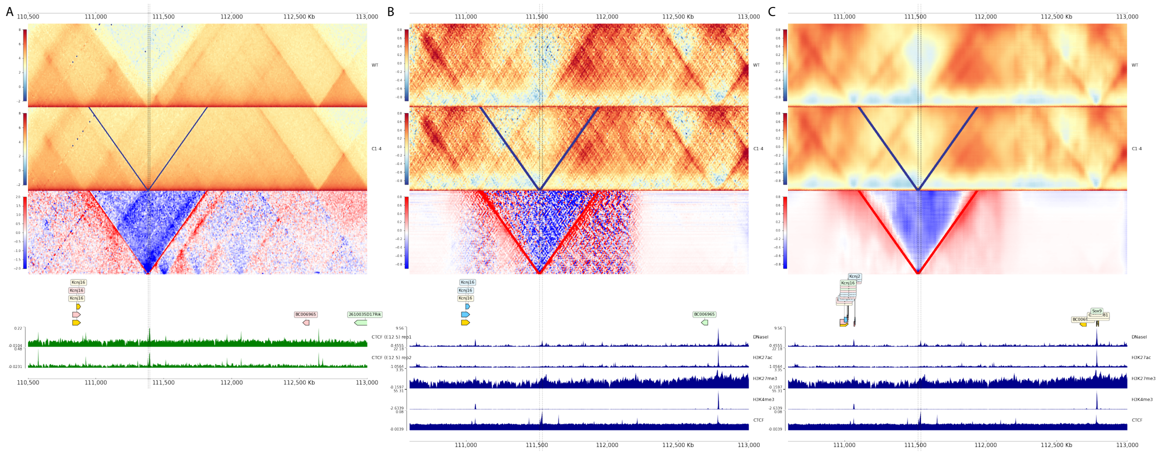

Supplementary Figure S14: Predicting 3D structural changes after CTCF deletion with the MSE+GAN and MSE-only models. **(A)** Ground truth Hi-C map for before perturbation (top), after perturbation (middle), and the absolute difference (bottom) **(B)** MSE+GAN model prediction for before and after perturbation. **(C)** MSE-only model prediction for before and after perturbation.
