# Supplementary material for: Epiphany: predicting Hi-C contact maps from 1D epigenomic signals
Source: Genome Biol. 2023 Jun 6;24:134. doi: 10.1186/s13059-023-02934-9 (PMC10242996; doi:10.1186/s13059-023-02934-9)
Supplement: Supplementary file 6 — Additional file 6. Review history. [file 13059_2023_2934_MOESM6_ESM.docx]

Review History

**First round of review**

**Reviewer 1**

**Were you able to assess all statistics in the manuscript, including the appropriateness of statistical tests used?** Yes.

**Were you able to directly test the methods?** Yes.

**Comments to author:**

Yang et al. developed Epiphany, a machine learning model to predict Hi-C contact maps using various epigenomic signals as input. Epiphany uses 5 types of epigenomic tracks to predict cell-type specific H-C contact maps. Compared to previous studies with similar architecture, the epigenetic inputs from different cell types enables Epiphany for cell type specific prediction. The Epiphany model has similar architecture as DeepC by replacing Dilated-CNN with an LSTM network and added a GAN loss for more realistic outputs.

Overall, this is an interesting study and the authors have performed multiple analyses to validate the accuracy of the presented models. However, there are also some serious concerns, including incremental improvements in prediction quality compared to previous studies like Akita and DeepC, addition of novel GAN loss that actually degrade model performance.

Major comments:

1. The introduction of a GAN module is interesting in the sense that it generates "realistic" (i.e. introducing noise) Hi-C contact maps. I believe that the motivation of the authors here is to enable the users to utilize the predicted Hi-C maps for downstream tasks. However, I am not sure that this will have broad applications to justify a drop in overall performance. Specifically, the downstream tasks after generating a contact matrix are usually limited to compartment and TAD identification. I doubt that any of these analyses will be affected by not making the contact matrices more "realistic". Other tasks, in particular loop calling, that may be affected by noise, are directly utilizing filtered reads as input.

2. In addition, adjusting prediction target like DeepC or downstream analysis pipeline might be a better approach than using GAN loss. Also in line 152, the correlation gap between Pearson (0.7028) and Spearman(0.5303) is quite large. Is this also due to the addition of noise pattern produced by GAN?

3. How can this method be used together with loop calling methods that operate directly on filtered read pairs and not binned read pairs?

4. The authors propose that Epiphany can be used to predict changes in 3D architecture upon disruption of the epigenetic marks used in the model. It would be very interested to test this hypothesis. For example, an EZH2 inhibitor can be used to disrupt H3K27me3. Epiphany can be used to predict the disrupted Hi-C and compare to ground truth. Similarly, HDAC inhibitors can be used to disrupt the H3K27ac signal. These type of experiments would be a very important experimental validation of the Epiphany model, which precisely focuses on these 3 key histone modifications. Additionally, such experiments will increase the impact of this work and highlight its clinical applications related to the use of epigenetic drugs, particularly in cancer.

5. Incremental structural prediction performance compared to previous models: In figure 2D, the predicted TADs are similar to the experiment, but many boundary locations are off. In Figure 3D, the comparison between Epiphany, Akita and ground truth is not convincing that Epiphany has better performance. Admittedly, the contrast and texture is much closer to the real value owing to the addition of adversarial loss. The improvement structural-wise is incremental. It would be more helpful if the author could point out structures that are missed by Akita but predicted by Epiphany. Also in Figure 6 A, B, the masking performance does not mimic the experimental Hi-C experiment. In Figure 6 C, it would be great to have a ground truth Hi-C after deletion on the side for comparison.

6. Large gap between training and testing performance: In Table 1, there seems to be a large gap between training and validation performance. The paper did not mention how many training data are generated. If the training data size is too small, model could overfit easily. To increase training data size, I would suggest reducing the overlapping window, and use data augmentations.

7. Genome wide cell type specific prediction performance: The author showed 8 loci of cell type specific predictions. It would be more interesting to see how Epiphany perform on the whole genome on predicting cell type specific Hi-C contact maps.

8. Comparison between Bi-LSTM and regular CNN SHAP score: Line 294, Figure 5C: I am not sure how CNN is replaced here. The process should be described in the method section. It seems like the author does not use a CNN with dilation or strides because saliency score outside of the output region is 0. Therefore, it cannot be compared with LSTM on SHAP score at that locus. Since the CNN model does not have access to that data, the SHAP score at the squared region will be 0. I suggest the author to run a dilated CNN or a strided CNN (or with pooling) to cover that region.

Minor comments:

1. Figure 2A: I noticed a consistent spike at the end of each trajectory, and I am not sure if that is an artifact of the calculation.

2. Line 114: The blurriness might be an effect of L2 loss. MSE is the solution to a MLE under the assumption that noise have gaussian distribution.

3. Line 234: I think it would make a stronger argument if the comparison is between the new two new cell types: H1 and K562.

4. Line 246: Bigwig tracks have noisy background, so I am not sure that choosing 0 as a SHAP baseline signal is the best approach.

5. Figure 6B: It might be a good idea to plot the difference between predictions, otherwise it is hard to see the merge event.

Typos:

1. Figures 3, 4, 5: The content for Figure 3, Figure 4, and Figure 5 are permuted in the wrong order.

2. Figure 1C: right side: Groun(d) Truth

3. Line 527: cell-type-specific structure(a)

**Reviewer 2**

**Were you able to assess all statistics in the manuscript, including the appropriateness of statistical tests used?** Yes.

**Were you able to directly test the methods?** No.

**Comments to author:**

This manuscript proposed a new method using ChIP-seq data for CTCF, H3K27ac, H3K27me3, H3K4me3 and DNase I hypersensitive sites to predict Hi-C maps. The authors tried to make the following claims: 1) its result is comparable to Akita; 2) it can make cell type specific prediction; 3) it can make predictions on a perturbed genome.

In general, the manuscript is in a well-organized and the results are clearly described. However, some examples in manuscript were not clear/convincing not significant enough or cannot serve the purpose. Some of the method descriptions are vague and need further improvement. Below are my detailed comments:

1. The order of the figure organizations is inconsistent with the main text. The correct read order for the figures should be Figure 1,2,4,5,3,6. However, for the convenience of the review, I still used the original figure order.

2. The current epiphany package from GitHub can't correctly run. Error message: AttributeError: 'torch.dtype' object has no attribute 'type';

3. The method section doesn't provide a clear description of how 1D epigenomic signal tracks were dissected. For example, how was the 3.4Mb length of input picked? Randomly selected from the genome or consecutively segmented across the genome?

4. In Figure 3A, the authors demonstrated that the five input feature tracks used here are somehow redundant as the model performs well without CTCF or DNaseI. A recent paper (https://www.biorxiv.org/content/10.1101/2022.03.05.483136v1) used only CTCF and ATAC-seq for cell type specific Hi-C prediction. I was wondering in this Epiphany method is it possible to use only CTCF and DNaseI to predict.

5. In figure 6C, the author tried to demonstrate that method can also predict Hi-C maps with deletion. However, there seems no ground truth for the prediction after deletion and it's hard to tell whether this prediction is correct. To claim that the method can predict genome perturbed Hi-C maps, the authors need to work on examples with real structure variations with known ground truth.

6. Figure 4C showed the Pearson correlation between Epiphany and Akita, but the author doesn't provide the Pearson correlation value and p-value.

7. In Figure 5A, the author used different targets for the same region in H1Esc and GM12878, which makes the comparison between the predictions difficult. Also, the structures at regions in this figure are quite similar in H1Esc and GM12878, and the signal intensity is the main difference. It would be better if the author could show predictions at more significant different structures.

8. Figure 2B/3B occurred before 2A/3A in the main text description.

9. Gene names under the Hi-C maps are cluttered together and unable to recognize.

**Authors Response**

**Response to reviews**

We thank the reviewers for their critiques and suggestions, which we have addressed in full in the revision. Please find a point-by-point response below. For convenience, all new results and analyses that have been added to the revised manuscript are also included as reviewer figures below.

**Reviewer #1**

*Yang et al. developed Epiphany, a machine learning model to predict Hi-C contact maps using various epigenomic signals as input. Epiphany uses 5 types of epigenomic tracks to predict cell-type specific H-C contact maps. Compared to previous studies with similar architecture, the epigenetic inputs from different cell types enables Epiphany for cell type specific prediction. The Epiphany model has similar architecture as DeepC by replacing Dilated-CNN with an LSTM network and added a GAN loss for more realistic outputs.*

*Overall, this is an interesting study and the authors have performed multiple analyses to validate the accuracy of the presented models. However, there are also some serious concerns, including incremental improvements in prediction quality compared to previous studies like Akita and DeepC, addition of novel GAN loss that actually degrade model performance.*

We thank the reviewer for finding our study to be interesting and our models well validated by multiple analyses. To clarify, the central contribution of the Epiphany model is the ability to make accurate cell-type-specific predictions of the Hi-C contact map from 1D epigenomic data. Since both Akita and DeepC learn to predict the contact map from genomic DNA, these models cannot be expected to generalize to new cell types. Indeed, while Akita and Epiphany perform comparably in the setting of held-out regions within a cell type, we show in the manuscript that Akita – which is trained in multi-task fashion across multiple cell types – fails to predict cell-type-specific structures, while Epiphany can generalize to new cell types and accurately predict cell-type specific contact maps. We have now extended these analyses to quantitatively show Epiphany’s advantage over Akita for cell-type-specific prediction (across regions held-out by Akita, see below). Therefore, Epiphany is not an incremental improvement over Akita and DeepC but rather a model that accomplishes the novel task of generalizable cell-type-specific contact map prediction.

We also disagree with the characterization of our novel GAN loss as degrading model performance. We make the case that MSE is the wrong loss to optimize: without the GAN loss, the MSE is improved but the predicted contact map is blurry and highly smoothed and perceptually has incorrect properties – e.g. bright regions are rounded rather than displaying the edges seen in true Hi-C contact maps – and therefore is difficult for humans to interpret. In fact, this “perception-distortion trade-off” has been addressed theoretically in the image restoration literature (Blau and Michaeli, *arxiv* 2020), where optimizing *any* distortion measure necessarily leads to reduced perceptual quality, and GANs are proposed as a principled way to approach the approach the perception-distortion bound.

To illustrate this idea, below (and in supplemental figures) we provide synthetic examples of perceptually poor Hi-C contact maps that achieve good MSE relative to ground truth (but poor GAN scores). Moreover, due to poor perceptual quality, it is difficult to use the MSE-only (“blurry”) maps for in silico perturbation experiments, such as predicting the outcome of deleting CTCF sites (see below). Nevertheless, since we train against HiC-DC+ processed observed/expected values, the blurry maps can be used to rank interactions and call loops and indeed do slightly better at this loop-calling task than the MSE+GAN maps, especially at greater genomic distance (full results below). Both MSE and MSE+GAN can be used for TAD calls, with highly correlated results (but a slight advantage to MSE-only). We have therefore updated the presentation in the text to discuss the improved perceptual quality of using MSE and GAN loss rather than simple MSE, which facilitates human interpretation and in silico perturbation experiments, while acknowledging that detection of the most significant interactions is somewhat improved in the MSE-only version of the model.

***Major comments:***

***1.	The introduction of a GAN module is interesting in the sense that it generates "realistic" (i.e. introducing noise) Hi-C contact maps. I believe that the motivation of the authors here is to enable the users to utilize the predicted Hi-C maps for downstream tasks. However, I am not sure that this will have broad applications to justify a drop in overall performance. Specifically, the downstream tasks after generating a contact matrix are usually limited to compartment and TAD identification. I doubt that any of these analyses will be affected by not making the contact matrices more "realistic". Other tasks, in particular loop calling, that may be affected by noise, are directly utilizing filtered reads as input.***

We address this comment at some length below since the usefulness of the GAN component of the model is an important claim in the paper. First, to illustrate why MSE alone is an inappropriate metric for evaluating the quality of the predicted contact map, we generate synthetic examples for which MSE is good but perceptually the contact map is poor; the GAN easily identifies these synthetic examples as not real Hi-C. We also show that when we threshold the predicted interactions from the “blurry” MSE-only model to obtain the most significant predicted interactions, we obtain blob-like significant regions of the contact map, while the ground truth and MSE+GAN model identify stripe-like significant regions. We link these observations to theoretical work on the perception-distortion trade-off from the image reconstruction literature, which implies that optimizing on MSE (or any distortion measure) alone will lead to perceptually poorer reconstruction.

When we train using observed/expected (O/E) values from HiC-DC+, we can indeed use both the MSE and MSE+GAN models for loop calling (or more precisely, for calling significant interactions). Therefore, the downstream tasks we envision are: (i) TAD calling, (ii) loop calling, and (iii) predicting the 3D consequences of epigenomic perturbations. (Note that we do not address compartment calls since we do not predict chromosome-by-chromosome contact matrices.) For (i), both MSE-only and MSE+GAN perform similarly, since the insulation score used for TAD boundary detection is a sum of a large number of “pixels'' from the contact map, however the MSE-only model produced insulation scores with slightly higher correlation to ground truth. For (ii), both models also perform equivalently for smaller genomic distances (<250kb), but it is true that at greater genomic distances where there are few true interactions, the GAN component somewhat degrades performance. For (iii), since the blurry MSE-only model predictions are perceptually poor and hard to interpret, we show that only the MSE+GAN model gives interpretable predictions of how the contact map changes under epigenomic changes like deletion of CTCF sites. Therefore, the GAN component has led to slight reduction in performance at downstream tasks of significant interaction and TAD calling, with the advantage of obtaining interpretable maps for predicting structural changes under epigenomic perturbations.

Overall, we believe these points justify the use of the GAN component in Epiphany. However, since TAD and significant interaction calling can be performed with the blurry MSE-only predictions (using O/E as the target when training), we have changed how we motivate the GAN in the text, and added the experiments into supplementary figures. Details on each of the points are provided below.

**Using MSE alone as a loss function is not adequate for learning Hi-C structures.** To demonstrate why MSE alone is not a good loss function for evaluating Hi-C structures, we show true, predicted and synthetic Hi-C maps for an example region on chromosome 3 in **Fig. R1** below. Each Hi-C map is rotated: the y-axis on the left shows the genomic coordinates, and the x-axis shows the distance from the diagonal. From left to right we plot: the ground truth Hi-C map, the prediction from MSE model, the prediction from GAN model, synthetic Hi-C map 1 (local average of the Hi-C map), synthetic Hi-C map 2 (local average for each genomic distance on the Hi-C map), synthetic Hi-C map 3 (averaged value at each genomic distance). The MSE value compared with ground truth, and the discriminator score calculated from the well-trained discriminator are shown in the subtitles.

We can see from **Fig. R1** that although synthetic maps look nothing like the real Hi-C map, they can still achieve a low MSE score compared to ground truth. For example, synthetic map 1 has equivalent MSE loss compared with the GAN prediction; synthetic map 2 has almost the same MSE loss as the blurry prediction. We conclude that MSE alone does not capture the quality of the contact map, and that optimizing MSE alone may not be enough to generate good predictions. We therefore added a GAN component to push the model to generate more realistic maps. Disc shows the score calculated from the well-trained discriminator, high score means the matrix is very Hi-C like, and low score indicates the matrix is not a valid Hi-C. The discriminator can easily tell that the synthetic matrices are not Hi-C matrices.

**Figure R1:** Ground truth, MSE-only (“blurry”) and GAN predictions, and synthetic maps, with MSE and GAN scores.

**MSE-only model gives perceptually incorrect regions of significant interactions.** To further explore the nature of the “blurry” version MSE-only model predictions, we examined the predicted regions of significant interactions in each case. In **Fig. R2-R4** below, we show random regions from test chromosome 3 of GM12878 (size: 2Mb along the diagonal, 1Mb from the diagonal). In each case, the left column shows the Hi-C map of ground truth (top), prediction from MSE-only trained model (middle), and prediction from the MSE+GAN model (bottom), using O/E target values. The middle column shows the significant interactions called from each map, where interactions >= 2 are marked as 1 (“significant”), and interactions < 2 are marked as 0 (“not significant”). The right column shows the absolute difference of the significance plot (green=0, no difference; yellow=1, false negatives, purple=-1, false positives).

In **Fig. R2**, there are 1496 falsely predicted bins in the MSE-only blurry prediction and 1815 falsely predicted bins in the GAN prediction (i.e. either false positives or false negatives). The MSE-only blurry prediction gives slightly better accuracy than the GAN prediction. However, if we check the spatial distribution of the significant calls, the GAN model produces better structure than the MSE-only model. We can see from the binarized interaction plot (middle column) that predictions from the MSE-only model give “blob-like” regions of significant interactions due to its blurriness, while the GAN model identifies edges and TAD-like structures. We also found that the false predictions from the GAN model were usually caused by a small pixel shift.

**Figure R2:** Binarized predictions show incorrect structure for MSE-only model.

The “blobby” spatial distribution of significant interactions predicted by the MSE-only model were common genome-wide. In another example region, there were 1003 falsely predicted bins for the MSE-only model and 1249 falsely predicted bins for the GAN model (**Fig. R3**). In this case, the MSE-only model detects a blob at the apex of the TAD but misses the edge structure.

**Figure R3:** Binarized predictions show incorrect structure for MSE-only model.

In a third example (**Fig. R4**), the MSE-only and MSE+GAN models had 1962 bins vs. 2007 false predictions. Again, the GAN model much more convincingly captures the TAD structure.

**Figure R4:** Binarized predictions show incorrect structure for MSE-only model.

**Perception-distortion trade-off.** The observations above that the MSE relative to ground truth can be good for a perceptually poor synthetic or predicted contact map (**Fig. R1**), and the perceptually apparent structural problems of the MSE-only binarized predictions (**Fig. R2-R4**) are related to the “perception-distortion trade-off” that has been described theoretically in the image reconstruction literature (Blau and Michaeli, *arxiv* 2020), where the model tries to reconstruct an image $\hat{X}$ from a degraded version the original image $X$. Briefly, the authors show that there is a trade-off between optimizing the distortion measure between the reconstructed image $\hat{X}$ and original image $X$ vs. improving the perceptual quality of $\hat{X}$, and a GAN provides a principled approach to navigate this trade-off.

**Comparison of predicted significant interactions.** We next assessed model performance at downstream tasks such as identifying significant interactions. We used the predicted contact maps for the MSE-only (“blurry”) trained model vs. the MSE+GAN model (with O/E target) to identify significant interactions, where true significant interactions were defined by ground HiC-DC+ z-score >= 3, and assessed the performance on held-out chromosomes. We found that both models performed well at this task, with the MSE-only slightly outperforming the MSE+GAN model (**Fig. R5**, auROC = 0.8858 vs. 0.8658, auPR = 0.4143 vs. 0.3110).

**Figure R5:** Performance of MSE-only (“blurry”) and MSE+GAN (“GAN”) models using O/E target for detection of significant interactions. True positives defined by ground truth HiC-DC+ Z-score of 3 or greater.

We next plotted the ROC curves for the two models when stratified by genomic distance (**Fig. R6**). In each plot, the light blue curve is the ROC for the MSE-only (“blurry”) model prediction, the dark blue curve for GAN prediction. Both models give similar performance at smaller genomic distances (under 250Kb), while the GAN prediction performance decreases as the genomic distance increases. This may be due to the artifacts caused by the GAN model at regions distant from the diagonal where the number of significant interactions is low.

**Figure R6:** Detection of significant interactions when stratified by genome distance. ROC curves for MSE-only (“blurry”) and MSE+GAN (“GAN”) models using O/E target, with true positives defined by ground truth HiC-DC+ Z-score of 3 or greater, for different genomic distance ranges.

**TAD calling evaluation via Pearson correlation of insulation scores.** We also compared TAD calling results for the MSE model vs. MSE+GAN model by computing the Pearson correlation of the insulation scores between the two predictions and the ground truth. Insulation scores were calculated on the three test chromosomes (chr3, 11, 17) by TopDom with five different window sizes covering from 100k to 500k distance from the diagonal. Outliers greater than 99.5% in the insulation score were removed during calculation. We found that both models perform well on the three test chromosomes, and the MSE blurry model performs slightly better (**Fig. R6**). The full table of Pearson correlation for all the chromosomes and window sizes has been appended in **Appendix Table 1** at the end.

**Figure R7:** Pearson correlation of insulation score on the three test chromosomes (chr3, 11, 17) of MSE only model vs. ground truth (blue), and MSE+GAN model vs. ground truth (red).

**MSE-only model fails to clearly predict 3D consequences of epigenomic perturbations.** We have further compared the ability of the MSE-only and MSE+GAN models to make interpretable predictions of 3D structural changes under epigenomic perturbations, in particle for the CTCF perturbation example. **Fig. R8** below shows the ground truth (left), MSE+GAN model prediction (middle) vs. MSE-only model prediction (right). In each panel, the top shows the contact map for WT, the middle shows the map after CTCF deletion, and the bottom shows the absolute difference between the two conditions. We can see that MSE+GAN prediction showed a clear TAD boundary disappearance after CTCF deletion, but the prediction from the MSE-only model less precisely shows the difference.

**Figure R8:** Predicting 3D structural changes after CTCF deletion with the MSE+GAN and MSE-only models.

**Evaluating predictions with the discriminator.** Since we train Epiphany with both MSE and adversarial loss, we can use the well-trained discriminator from the model as a perceptual score to evaluate predictions. In **Fig. R9** below, we plot the perceptual score for all sub-regions (200*100, 2Mb along the diagonal, 1M from the diagonal) predicted on chr3.

In the left panel of **Fig. R9**, the x-axis shows the genomic location of each 2Mb*1Mb sub-region along the diagonal and the y-axis shows the perceptual score. Blue dots show the perceptual score for ground truth Hi-C, green dots for the GAN prediction, and orange for the MSE-only prediction. All contact maps predicted by the MSE-only model obtain very low perceptual scores.

In the right panel of **Fig. R9**, we show example regions with the highest perceptual score in ground truth (A), MSE-only prediction (B) and GAN prediction (C); note that these examples correspond to different genomic locations.

**Figure R9.** Perceptual scores from the GAN for MSE-only and MSE+GAN model predictions.

***2.	In addition, adjusting prediction targets like DeepC or downstream analysis pipeline might be a better approach than using GAN loss. Also in line 152, the correlation gap between Pearson (0.7028) and Spearman (0.5303) is quite large. Is this also due to the addition of noise patterns produced by GAN?***

**DeepC adjustment of prediction targets vs GAN loss.** Please note that we do present a range of approaches for preprocessing the raw contact matrix, which amounts to an adjustment of the prediction targets. Epiphany showed consistently good prediction performance for various normalized targets: KR, ICE, and HiC-DC+ z-value and observed/expected count ratios. For the majority of our downstream applications, we used HiC-DC+ for preprocessing the contact matrix. HiC-DC+ uses a negative binomial GLM to estimate a background model and thus determine the significance of chromatin interactions. Using HiC-DC+ z-score or observed/expected count ratios as target values has the advantage that we can directly predict significant interactions by thresholding on the predicted interaction (“pixel”) value.

In the DeepC preprocessing method (**Fig. R9**, borrowed from the DeepC paper), the authors discretized the count values into distance-adjusted percentile bins as shown below (Schwessinger et al., 2020). The predicted contact maps can be used for visual inspection and calling TADs but cannot be used for significant loop calls.

**Figure R9.** DeepC preprocessing, reproduced from Schwessinger et al., 2020.

**Pearson vs. Spearman correlations on test chromosomes.** The version of Epiphany trained with ICE normalization did have a big gap between Pearson and Spearman correlation, but we do not agree that this gap was caused by the GAN component. **Table 1** with performance results from the main paper is reproduced below:

We note that this correlation gap only happened with ICE normalization but not for the models trained against other targets. All other models displayed a more modest ~0.05 drop from Pearson to Spearman correlation. This pattern was consistent between models with and without the GAN component (see Table 1 lambda=1 model, which uses MSE only). We also note that the Spearman correlation values for ICE normalization was similar to other models, but the Pearson correlations were very high, likely due to the smoothness of ICE normalization which makes the Pearson correlation task easier.

***3.	How can this method be used together with loop calling methods that operate directly on filtered read pairs and not binned read pairs?***

Since Epiphany predicts binned contact maps at fixed resolution (e.g. 5Kb, 10Kb), the predicted contact maps cannot be used with loop calling methods that operate on filtered read pairs.

***4.	The authors propose that Epiphany can be used to predict changes in 3D architecture upon disruption of the epigenetic marks used in the model. It would be very interesting to test this hypothesis. For example, an EZH2 inhibitor can be used to disrupt H3K27me3. Epiphany can be used to predict the disrupted Hi-C and compare it to ground truth. Similarly, HDAC inhibitors can be used to disrupt the H3K27ac signal. These types of experiments would be a very important experimental validation of the Epiphany model, which precisely focuses on these 3 key histone modifications. Additionally, such experiments will increase the impact of this work and highlight its clinical applications related to the use of epigenetic drugs, particularly in cancer.***

We agree this kind of global evaluation would be interesting, but we are not aware of any publicly available genome wide data that provides both Hi-C and 1D epigenomic inputs. Also, we would caution that massive global changes in histone marks (as opposed to local alterations) might in fact change the rules that govern the association between 1D and 3D organization. Indeed, one might want to explore how the model changes under global perturbations. We have added this caveat to the Discussion and leave the problem of modeling global alterations in 3D architecture to future work.

***5.	Incremental structural prediction performance compared to previous models: In figure 2D, the predicted TADs are similar to the experiment, but many boundary locations are off. In Figure 3D, the comparison between Epiphany, Akita and ground truth is not convincing that Epiphany has better performance. Admittedly, the contrast and texture is much closer to the real value owing to the addition of adversarial loss. The improvement structural-wise is incremental. It would be more helpful if the author could point out structures that are missed by Akita but predicted by Epiphany. Also in Figure 6 A, B, the masking performance does not mimic the experimental Hi-C experiment. In Figure 6 C, it would be great to have a ground truth Hi-C after deletion on the side for comparison.***

**TAD boundary location.** Boundary location variability is a common problem in TAD analysis and is due to lack of robustness in the change point detection. We can see from **Figure 2** in the main paper that among all ground truth Hi-C contact maps, the boundary locations vary between different normalization methods (dashed black vertical lines indicate the boundaries called from KR normalization). In our experience, the location of the change points is also not stable under other hyperparameter choices like Hi-C bin size and window size for the insulation score. This is why we report correlation of insulation scores instead of comparing the exact locations of TAD boundaries.

**Comparing Akita and Epiphany.** Our main claim about improving from Akita is to learn correct cell-type-specific structures. To expand this analysis, we examined the correlation between the ground truth and predicted structures for GM12878 and H1-hESC, as well as prediction of structural similarities/differences between H1-hESC and K562. These results are presented in **Fig. R12, R16** below and in **Supplementary Fig. S7, S9** in the paper.

Consistent with our findings, a recent preprint (Tan et al., *bioRxiv* 2022) presents a model called C.Origami that uses both DNA sequence and DNaseI + CTCF to predict Hi-C contact maps. In their paper, they showed ablation analysis that epigenomic tracks are of vital importance in making cell-type specific predictions.

In addition, we re-trained the Epiphany model with only DNaseI and CTCF tracks, and obtained good results (**Fig. R20**, **Supplementary Fig. 10**).

**Masking in Figure 6.** For **Figure 6 A,B**, we have changed the masking of the CTCF sites to an excision approach to better model the genomic perturbation, and we have added the results below (**Fig. R10** below and replacing the previous version of **Figure 6 A,B**). We only deleted one CTCF site (chr11:111520000-111540000), and compared the difference between the two conditions.

**Figure R10.** In silico prediction of effects of CTCF boundary site deletion using excision.

**Ground truth Hi-C for deletion.** We have added another epigenomic perturbation example where ground truth Hi-C data is available. [Wu et al.](https://www.nature.com/articles/s41467-021-24951-7) (2021) experimented with CTCF perturbation at the *SOX17* locus in human ES cells. **Fig. R11** (left) shows Capture Hi-C maps at the *SOX17* locus before (top) and after (bottom) targeting the CTCF site. The deleted region (marked in dashed vertical lines) is very close to the *SOX17* distal regulatory element (DRE). The authors found that removing the CTCF peak at the boundary upstream of the *SOX17* locus leads to decreased interaction of *SOX17* and its DRE, and increased interaction with the upstream boundary near the *RGS20* locus. **Fig. R11** (right) shows the Epiphany prediction at the *SOX17* region before (top) and after (bottom) CTCF deletion. We have **replaced the second biological example in Fig. 6 C,D** in the manuscript.

**Figure R11.** Perturbation of CTCF sites at the *SOX7* locus with ground truth Hi-C.

***6.	Large gap between training and testing performance: In Table 1, there seems to be a large gap between training and validation performance. The paper did not mention how much training data is generated. If the training data size is too small, the model could overfit easily. To increase training data size, I would suggest reducing the overlapping window, and use data augmentations.***

As we described in the Methods section, when training with GM12878 data, we used chromosomes 3, 11, 17 as completely held-out data for testing, and all the other chromosomes as training data. Epiphany only has 4 convolutional layers, 3 bi-LSTM and 2 fully-connected layers, and is relatively light-weight compared to current Hi-C prediction models (e.g. DeepC, Akita). We think having 19 chromosomes as training as 3 as testing is a valid split.

***7.	Genome wide cell type specific prediction performance: The author showed 8 loci of cell type specific predictions. It would be more interesting to see how Epiphany performs on the whole genome on predicting cell type specific Hi-C contact maps.***

Thank you for this suggestion. We evaluated the genome-wide cell-type specific prediction performance at two levels. First, we added systematic analyses across all the held-out test regions of the Akita model: the cell-type specific comparison with consistent targets (Akita’s normalization method). The results are shown in **Fig. R12** (and **Supplementary Fig. S6**), which provides the Pearson correlation of 42 testing regions of GM12878 (left) and H1-hESC (right). The x-axis shows the correlation between ground truth and prediction using the Akita model, and the y-axis shows the correlation using Epiphany. For H1-specific prediction – to provide a fair comparison with Akita – we retrained the Epiphany model using the training chromosomes in H1-hESC, so that the Epiphany predictions in the both figures are cross-chromosomal but within cell type. (Due to the sparseness of the ground truth H1-hESC data, the H1 model was trained with MSE-only loss). We found that Epiphany gave slightly better prediction performance compared with Akita in both GM12878 and H1-hESC.

**Figure R12.** Quantitative comparison of Epiphany and Akita on H1ESC on held-out regions on 3 chromosomes.

Second, similar to the performance evaluation on test chromosomes in GM12878 (**Figure 3**), we also evaluated the prediction performance on all test chromosomes in K562 (cross-cell type, cross-chromosomal prediction). In the scatter plot in **Fig. R13 and Supplementary Fig. S7**, each dot corresponds to a 2Mb along the diagonal by 1Mb from the diagonal region, colored by chromosome. The x-axis shows the average MSE loss between Epiphany’s prediction vs. ground truth, and the y-axis shows the Pearson correlation of the insulation score in this region. The majority of the regions have a Pearson correlation higher than 0.5, and one good and bad example of predicted regions are shown on the right.

**Figure R13.** MSE and Pearson correlation for Epiphany predictions on held-out regions on 3 chromosomes in K562.

Below (**Fig. R14**) is a region with cell-type specific 3D structure in GM12878, K562 and H1-hESC (chr3:188,250,000-190,250,000). Ground truth Hi-C maps are shown on the left, and Epiphany predictions are shown on the right.

**Figure R14.** Cell-type-specific Epiphany predictions across GM12878, K562, and H1-hESC.

Finally, we found another example of a cell-type specific region around the gene *CPED1* ([Cai et al., 2021](https://www.nature.com/articles/s41467-021-20940-y)), and we tested whether Epiphany could predict the difference. The *CPED1* locus is covered by an H3K27me3 rich region in GM12878 but overlaps with a super-enhancer in K562 (shown in **Fig. R13**, left). The right of **Fig. R13** demonstrates the cell-type specific the prediction performance of Epiphany: (top to bottom) GM12878 ground truth structure, GM12878 contact map predicted by Epiphany; K562 ground truth, K562 contact map predicted by Epiphany; gene annotations; and epigenomic tracks (H3K27ac, H3K27me3) for the two cell types.

**Figure R15.** Epigenomic perturbation example using Epiphany models trained on GM12878 and K562.

***8.	Comparison between Bi-LSTM and regular CNN SHAP score: Line 294, Figure 5C: I am not sure how CNN is replaced here. The process should be described in the method section. It seems like the author does not use a CNN with dilation or strides because saliency score outside of the output region is 0. Therefore, it cannot be compared with LSTM on SHAP score at that locus. Since the CNN model does not have access to that data, the SHAP score at the squared region will be 0. I suggest the author run a dilated CNN or a strided CNN (or with pooling) to cover that region.***

This is a very good suggestion, and we agree that there are different structures that can cover bigger receptive fields and capture the contribution of distal elements. Due to the nature of the Hi-C contact map and the dependencies between nearby stripes on the map, we believe that the bi-LSTM layer is a natural choice. However, we now mention in the Discussion that other architectures like dilated CNNs are an option for capturing long range dependencies.

***Minor comments:***

***1.	Figure 2A: I noticed a consistent spike at the end of each trajectory, and I am not sure if that is an artifact of the calculation.***

Yes, this is an important observation, and we have checked our prediction and realized that this spike is caused by an artifact of prediction at the edges of the map. In practice, a smaller region (strictly smaller than 1Mb from the diagonal) of the predicted contact map should be used in downstream analyses to avoid this edge effect, or the model architecture should be slightly altered to predict a wider (>1 Mb from the diagonal) region to push the edge beyond the desired field of view. We have noted these issues in the manuscript.

***2.	Line 114: The blurriness might be an effect of L2 loss. MSE is the solution to a MLE under the assumption that noise has a gaussian distribution.***

We agree that the blurriness is expected given the underlying noise model assumption for MSE.

***3.	Line 234: I think it would make a stronger argument if the comparison is between the two new cell types: H1 and K562.***

In the original comparison, we chose H1 vs. GM12878 instead of K562 because we hoped to compare the cell-type specific prediction performance between Epiphany and Akita. Since the K562 cell line is not one of the multi-task training targets, Akita can not make the prediction for it. Therefore we chose H1-hESC and GM12878, which are both included in Akita’s training cell types. We have added a systematic analysis between H1-hESC and GM12878 predictions with Akita and Epiphany, shown in **Fig. R12**, where we have trained a new H1 model. We found that Epiphany gives slightly better cell-type specific predictions in these two cell types.

To address the reviewer’s suggestion, we have now added the K562 cell line into our comparison. In this analysis, we used the Epiphany model trained on GM12878 training chromosomes (all chromosomes except for 3, 11, 17) and compared its performance on H1-hESC and K562 chromosome 3. We first chopped the chromosome into 2Mb regions along the diagonal and calculated the Pearson correlation of insulation scores between H1-hESC and K562 from ground truth contact maps for each cell type. We then calculated the Pearson correlation of insulation scores from the predicted maps of the two cell lines. The results are shown in **Fig. R16**, where we plot the Pearson correlation of insulation scores between H1-hESC vs. K562 based on ground truth contact maps (x-axis) vs. predicted contact maps (y-axis). We have added these experiments in **Supplementary Figure S8**.

**Figure R16.** Epiphany prediction of insulation score correlation between cell types (H1-hESC vs. K562) vs. ground truth insulation score correlation.

We then ranked the 2Mb regions based on their insulation score correlation and checked several cell-type specific predictions visually. Two cell-type specific regions (top: chr3:78705000-80705000, and bottom: chr3:705000-2705000) are shown in **Fig. R17** below: on the left are the ground truth contact maps between K562 and H1-hESC, and on the right are the predicted contact maps between the two cell lines.

**Figure R17.** Examples of Epiphany cell-type-specific predicted contact maps.

***4.	Line 246: Bigwig tracks have noisy backgrounds, so I am not sure that choosing 0 as a SHAP baseline signal is the best approach.***

The reason that we initially used a zero baseline in our SHAP analysis was to see the influence of all the signals including the noisy background. We hoped to make sure that even with the noisy background, the Epiphany model could still use the real signals to make the prediction. The SHAP value calculations showed that Epiphany used biologically plausible signals from different epigenomic tracks to make the prediction. For convenience, the feature attribution results with zero background as reference are shown in **Fig. R18** below (and in the previous **Figure 4** in the manuscript).

**Figure R18.** DeepSHAP analysis using zero signal as reference.

To address the reviewer’s comment, we have added the SHAP value calculation using the averaged noisy background as baseline, with results shown in **Fig. R19**. The signals that contribute to the differential structure between GM12878 (left) and K562 (right) are very consistent with our previous results calculated using a zero background as reference. (Since the high similarity between the two predictions, we have kept the original SHAP value calculation using zero background in the manuscript.)

**Figure R19.** DeepSHAP analysis using average background signal as reference.

***5.	Figure 6B: It might be a good idea to plot the difference between predictions, otherwise it is hard to see the merge event.***

This is a very good idea, and we have added plots of the difference between contact maps before and after perturbation for both the predicted and ground truth contact maps.

***Typos:***

***1.	Figures 3, 4, 5: The content for Figure 3, Figure 4, and Figure 5 are permuted in the wrong order.***

We have fixed the figure.

***2.	Figure 1C: right side: Groun(d) Truth***

We have fixed the figure.

***3.	Line 527: cell-type-specific structure(a)***

We have fixed the text.

**Reviewer #2**

***This manuscript proposed a new method using ChIP-seq data for CTCF, H3K27ac, H3K27me3, H3K4me3 and DNase I hypersensitive sites to predict Hi-C maps. The authors tried to make the following claims: 1) its result is comparable to Akita; 2) it can make cell type specific prediction; 3) it can make predictions on a perturbed genome.***

***In general, the manuscript is well-organized and the results are clearly described. However, some examples in the manuscript were not clear/convincing, not significant enough or cannot serve the purpose. Some of the method descriptions are vague and need further improvement. Below are my detailed comments:***

We thank the reviewer for the comments below, and we have added additional method description to improve clarity as well as new results to provide more clear and convincing evidence of model performance.

***1.	The order of the figure organizations is inconsistent with the main text. The correct read order for the figures should be Figure 1,2,4,5,3,6. However, for the convenience of the review, I still used the original figure order.***

We have corrected the out-of-order figure references.

***2.	The current epiphany package from GitHub can't correctly run. Error message: AttributeError: 'torch.dtype' object has no attribute 'type';***

We believe the code issue has been resolved. The dtype bug was from a truncated data set in our training pipeline. We have replaced this data set and tested the pipeline; we have also provided a colab notebook for downstream analyses.

***3.	The method section doesn't provide a clear description of how 1D epigenomic signal tracks were dissected. For example, how was the 3.4Mb length of input picked? Randomly selected from the genome or consecutively segmented across the genome?***

We apologize for this oversight and have added more description on the model implementation:

“For each 1Mb stripe that is orthogonal to the diagonal on the Hi-C map, we take in a 1.4Mb window of epigenomic signal centered at this stripe as input (1Mb covers the corresponding interaction bins of the stripe, with an additional 400Kb on both sides of the input). Since we are predicting 200 consecutive stripes simultaneously, the total input region is 3.4Mb using an overlapping sliding window approach, where the window size is 1.4Mb for a single stripe and the step size is 10kb. Therefore, for each 200 stripe prediction, we will have our input as a 3D tensor with size (5 tracks x 3.4 Mb x 200 windows).”

The window size of 1.4 Mb for a single stipe (3.4Mb for 200 stripes) was selected by a grid search: we experimented with 1Mb (no additional signals), 1.2Mb, 1.4Mb, and 1.6Mb and found that 1.4 yielded good prediction, while further extending the input signals did not help the model performance. So we chose 1.4Mb as the window size.

***4.	In Figure 3A, the authors demonstrated that the five input feature tracks used here are somehow redundant as the model performs well without CTCF or DNaseI. A recent paper (***[***https://www.biorxiv.org/content/10.1101/2022.03.05.483136v1***](https://www.biorxiv.org/content/10.1101/2022.03.05.483136v1)***) used only CTCF and ATAC-seq for cell type specific Hi-C prediction. I was wondering in this Epiphany method is it possible to use only CTCF and DNaseI to predict.***

This is a good suggestion and a very interesting experiment. We have now added new performance results for a version of the Epiphany model trained only on CTCF and DNaseI tracks. We note that the main C.Origami model cited by the reviewer uses not only CTCF+ATAC but also DNA sequence, and therefore C.Origami may be less likely to generalize well across cell types.

We re-trained Epiphany using only DNaseI data and CTCF track, and the prediction performance is provided in **Table 1** (repeated from the main manuscript) and **Table R1** below. Our full model uses 5 epigenomic tracks: DNaseI, H3K27ac, H3K27me3, H3K4me3 and CTCF. In our ablation analysis, we found that removing DNaseI during training did not hinder the prediction performance. Comparing the full model with the DNaseI absent model using feature attribution analysis, we found that peaks from histone modification tracks strongly contributed to prediction performance when DNaseI was removed. We then suggested that there may be redundancies between the information from chromatin accessibility and histone modifications. The Pearson and Spearman correlations of different normalization approaches using the full model is shown below (**Table 1** in the manuscript).

Consistent with our previous observation and the C.Origami paper, we observed fairly good prediction performance using only DNaseI and CTCF tracks, as shown in **Table R2**.

|  | Training chromosomes | Testing chromosomes |
| --- | --- | --- |
| Pearson correlation | 0.5789 | 0.4638 |
| Spearman correlation | 0.5292 | 0.4224 |

**Table R1:** Performance result for Epiphany trained only on DNaseI+CTCF.

**Fig. R20** (also **Supplementary Fig. 9**) provides a visual comparison of ground truth GM12878 (top), prediction with the full Epiphany model (middle), and prediction with the DNaseI+CTCF model (below).

**Figure R20.** Comparison of full Epiphany model vs. Epiphany trained on DNaseI + CTCF.

***5.	In figure 6C, the author tried to demonstrate that this method can also predict Hi-C maps with deletion. However, there seems no ground truth for the prediction after deletion and it's hard to tell whether this prediction is correct. To claim that the method can predict genome perturbed Hi-C maps, the authors need to work on examples with real structure variations with known ground truth.***

We have added another application example (please see **Fig. R11** above) where ground truth Hi-C is available. [Wu et al.](https://www.nature.com/articles/s41467-021-24951-7) (2020) experimented the CTCF perturbation at *SOX17* locus in human ES cells. **Fig. R11** (left) shows Capture Hi-C data at the *SOX17* locus before CTCF (top) and after (bottom) CTCF deletion. The deleted region (marked in dashed vertical lines) is very close to the *SOX17* distal regulatory element (DRE). The authors found that removing the CTCF peak at the boundary upstream of the *SOX17* locus leads to decreased interaction of *SOX17* and its DRE and increased interaction with the upstream boundary near the *RGS20* locus. **Fig. R11** (right) shows the Epiphany prediction of the *SOX17* region before (top) and after (bottom) the CTCF deletion.

***6.	Figure 4C showed the Pearson correlation between Epiphany and Akita, but the author doesn't provide the Pearson correlation value and p-value.***

The Pearson correlation and significance (p-value) are included as **Appendix 1** at the end of this file and as **Supplementary Table S1** in the paper.

***7.	In Figure 5A, the author used different targets for the same region in H1Esc and GM12878, which makes the comparison between the predictions difficult. Also, the structures at regions in this figure are quite similar in H1Esc and GM12878, and the signal intensity is the main difference. It would be better if the author could show predictions at more significant different structures.***

We are limited in our choice of examples, since Akita only leaves our 300 regions genome wide as a test set; however we have included a systematic comparison on test regions that overlap our test chromosomes of the predictions of Akita and Epiphany for GM12878 and H1-hESC, and the predicted insulation score similarity between H1-hESC and K562. Please see **Fig. R12**, **R16** above (and **Supplementary Fig. S6** and **S8** in the paper).

**Limited regions for comparison between Akita and Epiphany.** In the comparison between Akita and Epiphany, we could only compare on a very limited set of regions. In the multi-tasking training scheme, Akita only left out 413 ~1Mb regions genome-wide as a test set (on the same chromosomes as the training set examples), and among them, only 42 regions overlapped with our test chromosomes (chr3, 11 and 17). Therefore, unfortunately, we could only perform the method comparison on these 42 regions.

**Comparing two models using different ground truth normalization**. For the majority of our downstream analyses with the Epiphany model, we used HiC-DC+ observed/expected count ratio and z-score normalization due to their convenience for significant interaction calling. In the cell-type specific comparison, we continued using the model trained with this normalization method, and showed the cell-type specific comparisons in **Figure 5**.

We agree that the comparison between two models using different normalized targets is difficult. We have now added the cell-type specific comparison with consistent targets (Akita’s normalization method). The results are in **Fig. R12** (and **Supplementary Fig. S6**) and show the Pearson correlation of t42 test regions in GM12878 (left) and H1-hESC (right). The x-axis shows the correlation between ground truth and prediction using the Akita model, and the y-axis shows the correlation using Epiphany. The Epiphany model is trained using training chromosomes in H1-hESC, so that the Epiphany predictions in the both panels are cross-chromosomal. (Due to the sparseness of the ground truth H1-hESC data, the H1 model is trained with MSE-only loss). We can see that Epiphany shows slightly better prediction performance compared with Akita in both GM12878 and H1-hESC.

*8.	Figure 2B/3B occurred before 2A/3A in the main text description.*

We have fixed the figure order issues.

*9.	Gene names under the Hi-C maps are cluttered together and unable to recognize.*

The older version showed all refGene annotations, and the cluttering is usually caused by inclusion of isoforms of the same genes. We have filtered out some unnecessary small genes to avoid the clutter, and in some cases we kept only single transcript isoforms for a clear visualization.

**Appendix 1: Pearson and p-value for comparing Akita and Epiphany model for GM12878**

| **Region** | **Akita_pearson2k** | **Akita_pearson** | **Akita_pvalue** | **Epiphany_pearson** | **Epiphany_pvalue** | **chr** |  |
| --- | --- | --- | --- | --- | --- | --- | --- |
| **0** | chr11:72808448-73857024 | 0.814081 | 0.821105 | 0.000000e+00 | 0.671717 | 0.000000e+00 | chr11 |
| **1** | chr3:188727296-189775872 | 0.552668 | 0.557439 | 0.000000e+00 | 0.789731 | 0.000000e+00 | chr3 |
| **2** | chr3:184532992-185581568 | 0.769163 | 0.781513 | 0.000000e+00 | 0.481597 | 0.000000e+00 | chr3 |
| **3** | chr17:51787776-52836352 | 0.303389 | 0.316284 | 0.000000e+00 | 0.519796 | 0.000000e+00 | chr17 |
| **4** | chr3:138702848-139751424 | 0.664819 | 0.674151 | 0.000000e+00 | 0.570447 | 0.000000e+00 | chr3 |
| **5** | chr11:75954176-77002752 | 0.382857 | 0.391371 | 0.000000e+00 | 0.336787 | 0.000000e+00 | chr11 |
| **6** | chr3:137129984-138178560 | 0.508738 | 0.522677 | 0.000000e+00 | 0.550651 | 0.000000e+00 | chr3 |
| **7** | chr3:185057280-186105856 | 0.554827 | 0.563293 | 0.000000e+00 | 0.470411 | 0.000000e+00 | chr3 |
| **8** | chr11:74905600-75954176 | 0.411526 | 0.419027 | 0.000000e+00 | 0.576363 | 0.000000e+00 | chr11 |
| **9** | chr17:52312064-53360640 | 0.034078 | 0.035423 | 2.070935e-15 | 0.460069 | 0.000000e+00 | chr17 |
| **10** | chr17:50739200-51787776 | 0.424608 | 0.429114 | 0.000000e+00 | 0.027292 | 7.174214e-09 | chr17 |
| **11** | chr3:188203008-189251584 | 0.386076 | 0.402455 | 0.000000e+00 | 0.690841 | 0.000000e+00 | chr3 |
| **12** | chr3:136081408-137129984 | 0.525553 | 0.536295 | 0.000000e+00 | 0.583120 | 0.000000e+00 | chr3 |
| **13** | chr11:77527040-78575616 | 0.713225 | 0.726106 | 0.000000e+00 | 0.600855 | 0.000000e+00 | chr11 |
| **14** | chr3:186630144-187678720 | 0.648759 | 0.653436 | 0.000000e+00 | 0.649324 | 0.000000e+00 | chr3 |
| **15** | chr11:73332736-74381312 | 0.535620 | 0.545981 | 0.000000e+00 | 0.373203 | 0.000000e+00 | chr11 |
| **16** | chr3:186105856-187154432 | 0.696271 | 0.704533 | 0.000000e+00 | 0.552614 | 0.000000e+00 | chr3 |
| **17** | chr3:139227136-140275712 | 0.502819 | 0.516174 | 0.000000e+00 | 0.621969 | 0.000000e+00 | chr3 |
| **18** | chr11:72284160-73332736 | 0.760599 | 0.764385 | 0.000000e+00 | 0.526985 | 0.000000e+00 | chr11 |
| **19** | chr11:78051328-79099904 | 0.830855 | 0.835785 | 0.000000e+00 | 0.505805 | 0.000000e+00 | chr11 |
| **20** | chr17:48117760-49166336 | 0.656065 | 0.660150 | 0.000000e+00 | 0.542893 | 0.000000e+00 | chr17 |
| **21** | chr3:187678720-188727296 | 0.319231 | 0.331149 | 0.000000e+00 | 0.572779 | 0.000000e+00 | chr3 |
| **22** | chr17:49166336-50214912 | 0.611288 | 0.618806 | 0.000000e+00 | 0.557226 | 0.000000e+00 | chr17 |
| **23** | chr11:76478464-77527040 | 0.344937 | 0.353284 | 0.000000e+00 | 0.612292 | 0.000000e+00 | chr11 |
| **24** | chr3:135557120-136605696 | 0.676867 | 0.683960 | 0.000000e+00 | 0.353466 | 0.000000e+00 | chr3 |
| **25** | chr3:136605696-137654272 | 0.819603 | 0.828199 | 0.000000e+00 | 0.690045 | 0.000000e+00 | chr3 |
| **26** | chr3:139751424-140800000 | 0.271358 | 0.283838 | 0.000000e+00 | 0.637182 | 0.000000e+00 | chr3 |
| **27** | chr11:71759872-72808448 | 0.614986 | 0.626514 | 0.000000e+00 | 0.516861 | 0.000000e+00 | chr11 |
| **28** | chr3:185581568-186630144 | 0.798682 | 0.804858 | 0.000000e+00 | 0.707656 | 0.000000e+00 | chr3 |
| **29** | chr11:73857024-74905600 | 0.504539 | 0.516394 | 0.000000e+00 | 0.148038 | 1.432485e-218 | chr11 |
| **30** | chr17:47593472-48642048 | 0.351323 | 0.354471 | 0.000000e+00 | 0.724229 | 0.000000e+00 | chr17 |
| **31** | chr17:50214912-51263488 | 0.596643 | 0.606685 | 0.000000e+00 | -0.006433 | 1.726206e-01 | chr17 |
| **32** | chr17:49690624-50739200 | 0.479496 | 0.488686 | 0.000000e+00 | 0.293161 | 0.000000e+00 | chr17 |
| **33** | chr3:137654272-138702848 | 0.429932 | 0.439299 | 0.000000e+00 | 0.482046 | 0.000000e+00 | chr3 |
| **34** | chr11:74381312-75429888 | 0.427185 | 0.434581 | 0.000000e+00 | 0.366883 | 0.000000e+00 | chr11 |
| **35** | chr3:187154432-188203008 | 0.868126 | 0.874793 | 0.000000e+00 | 0.821522 | 0.000000e+00 | chr3 |
| **36** | chr3:138178560-139227136 | 0.757415 | 0.765530 | 0.000000e+00 | 0.757154 | 0.000000e+00 | chr3 |
| **37** | chr17:48642048-49690624 | 0.228593 | 0.231955 | 0.000000e+00 | 0.455507 | 0.000000e+00 | chr17 |
| **38** | chr11:77002752-78051328 | 0.730736 | 0.738601 | 0.000000e+00 | 0.597457 | 0.000000e+00 | chr11 |
| **39** | chr11:78575616-79624192 | 0.149556 | 0.154212 | 1.351554e-264 | 0.470647 | 0.000000e+00 | chr11 |
| **40** | chr17:51263488-52312064 | 0.430880 | 0.445977 | 0.000000e+00 | 0.461802 | 0.000000e+00 | chr17 |
| **41** | chr11:75429888-76478464 | 0.491776 | 0.497861 | 0.000000e+00 | 0.691817 | 0.000000e+00 | chr11 |

**Appendix 2:** Pearson correlation of insulation score of the MSE only model and MSE+GAN model vs. ground truth.

|  | **chrom** | **window_size** | **Pearson_MSE** | **Pearson_GAN** |
| --- | --- | --- | --- | --- |
| **0** | chr3 | 10 | 0.8484910367253652 | 0.8367076007972397 |
| **1** | chr3 | 20 | 0.8326588035516042 | 0.8262635123894972 |
| **2** | chr3 | 30 | 0.8235818668003793 | 0.8168088668694704 |
| **3** | chr3 | 40 | 0.8169868080762421 | 0.8048275199432582 |
| **4** | chr3 | 50 | 0.8050651971282021 | 0.7878168921863402 |
| **5** | chr11 | 10 | 0.8290364517764348 | 0.8209635071211747 |
| **6** | chr11 | 20 | 0.8078767838021246 | 0.7927883919419553 |
| **7** | chr11 | 30 | 0.7912096768035607 | 0.7713723118349947 |
| **8** | chr11 | 40 | 0.7762045925015326 | 0.7554578477051357 |
| **9** | chr11 | 50 | 0.7521086583199597 | 0.7313809388017539 |
| **10** | chr17 | 10 | 0.7198135064550346 | 0.7107730034672004 |
| **11** | chr17 | 20 | 0.6530155615319198 | 0.6303006734584008 |
| **12** | chr17 | 30 | 0.45776899269134486 | 0.4494683592125574 |
| **13** | chr17 | 40 | 0.27977145465335457 | 0.28066565966964263 |
| **14** | chr17 | 50 | -0.007603171777556861 | -0.0011591862622279112 |

**Second round of review**

**Reviewer 1**

The authors have performed several computational experiments to further benchmark their models. Overall, based on their evaluation, it is clear the MSE loss performs better at downstream tasks (especially loop calling) compared to the proposed model (GAN loss), despite the authors’ initial claim that the GAN loss model was designed to improve downstream analysis. GAN loss may seem to generate more “realistic” images, but there is no quantifiable metric for this and at the end of the day what really matters is the quantifiable performance at downstream tasks where MSE loss proved to perform better.

Major comments:

1. It seems that Epiphany cannot predict some very obvious loops and domains, such as panels in Figure 2D, 3D, 6A-B. This is surprising given all the epigenomic signals that are included for training, and raises concerns for the model’s applications in a real setting. Can the authors show all the epigenomic tracks at these loci and provide explanations as to why the model is not able to make these predictions correctly?

2. The authors argue that the boundary regions change according to different normalization methods. However, it seems that TAD calling results relying on significance (p-value or q-value) are conserved, and those relying on obs/exp matrices are also consistent, as demonstrated in Fig. 2d-left (ground truth data). However, predictions from Epiphany seem to have frequent shifts at boundary locations.

3. I am not sure I agree with the authors that the goal should be to produce photorealistic images. In my opinion, it is more important to reveal biological insights which are measured by metrics like loop and TAD calling. MSE is obviously not a perfect objective function, but, as demonstrated in the authors’ responses to the first review, the corresponding MSE-only model clearly outperforms the proposed model (MSE+GAN). Thus, it is unclear why the focus should be on "perception" that cannot be quantified and is less biologically relevant and quite possibly noisier.

4. For the 42 loci tested in Figure R12, the author mentions that Epiphany prediction is “slightly better” when compared to Akita. However, it is difficult to tell the difference. We are not sure if Epiphany is capable of producing cell type-specific prediction. Also, for cell type-specific prediction, it seems that the authors had to actually train using MSE loss because of the lower sequencing depth of the H1 data, similar to longer range interactions. This is consistent with the concern that GAN loss introduced artificial noise which further undermined cell type-specific prediction performance.

5. Based on the authors' results, GAN loss does not perform well on long-range loops: the authors provide an explanation related to the lower number of reads at longer distances; again this argues in favor of the denoising properties on MSE. This also raises another concern: what is the minimum sequencing depth required for GAN loss to perform well in terms of loop calling at a given distance? The fact that for the H1 cell line the authors had to resort to using the MSE loss is additional evidence that the MSE model is better.

Minor comments:

1. To convert predicted Hi-C matrix to read pairs for loop calling, the authors could first convert the intensity s between location i and j to integers int(S) and generate int(S) number of lines of i and j pseudo interaction reads in the read pair files.

2. For comparison between CNN and LSTM, the author could use a larger CNN input range. We suggest either removing Figure 5C or run the comparison between CNN and LSTM with the same input window.

3. Can the author provide the code they used to generate the quantitative evaluation figures?

4. Human gene name, e.g. SOX17, should be all capital letters. Errors include Sox17.

**Authors Response**

**Point-by-point responses to the reviewers’ comments:**

*Reviewer #1: The authors have performed several computational experiments to further benchmark their models. Overall, based on their evaluation, it is clear the MSE loss performs better at downstream tasks (especially loop calling) compared to the proposed model (GAN loss), despite the authors' initial claim that the GAN loss model was designed to improve downstream analysis. GAN loss may seem to generate more "realistic" images, but there is no quantifiable metric for this and at the end of the day what really matters is the quantifiable performance at downstream tasks where MSE loss proved to perform better.* 

We disagree that producing realistic contact maps is unimportant, and as we explained in the previous response, the GAN discrimination score itself is a quantifiable metric for how realistic the map is (see also **Supplementary Fig. S1**, included above). However, to address the reviewer’s key concern, we now present Epiphany as a model with two versions, MSE-only and MSE+GAN, and the user can choose which one to use depending on whether downstream computation or visualization is more important.

*Major comments:
1. It seems that Epiphany cannot predict some very obvious loops and domains, such as panels in Figure 2D, 3D, 6A-B. This is surprising given all the epigenomic signals that are included for training, and raises concerns for the model's applications in a real setting. Can the authors show all the epigenomic tracks at these loci and provide explanations as to why the model is not able to make these predictions correctly?*

If you look at the Akita predictions on held-out test examples vs. ground truth in **Fig. 3D** (see Akita predictions from this panel in the figure below, together with Akita-processed ground truth), in at least 3/7 (arguably 4/7) cases, the predicted structure has very obvious mistakes (loops/domains missing or added), and other examples have smaller mistakes. (Additional examples of Akita predictions vs. ground truth can be seen in **Fig. 4A**, again showing incorrect predicted structure.) The nature of the problem is that it is difficult. We now include all epigenomic tracks in additional supplementary figures (please see **Supplementary Fig. S4** for the predictions in **Fig. 2D**, and **Supplementary Fig. S14** for **Fig. 6A** and **B**) as requested. However, please note that we are achieving performance comparable to the state-of-the-art Akita model while enabling generalization to a new cell type.

**Related to Figure 3D (revised manuscript)**. Top row: ground truth for 7 Akita test regions, using Akita pre-processing; bottom row: Akita predictions. Error in TAD or loop calls for state-of-the-art Akita model, left to right: (i) incorrect TAD structure, with small central subTAD and interactions missing; (ii) missing interactions/incorrect TAD structure; (iii) spurious long range interactions that are absent in ground truth; (iv) mostly correct structure; (v) missing interactions/stripes but qualitatively similar TAD structure; (vi) strong spurious interactions, incorrect TAD/subTAD structure; (vii) largely correct TAD structure but spurious stripes in bottom TAD that are not found in ground truth. Qualitatively, predictions for (i), (ii), (vi) and arguably (iii) have incorrect TAD/overall structure.

*2. The authors argue that the boundary regions change according to different normalization methods. However, it seems that TAD calling results relying on significance (p-value or q-value) are conserved, and those relying on obs/exp matrices are also consistent, as demonstrated in Fig. 2d-left (ground truth data). However, predictions from Epiphany seem to have frequent shifts at boundary locations.*

The TAD caller we are using (TopDom) is a change point analysis method where the boundary is called at the trough (min value) of the moving average curve shown (**Fig. 2D**). In both the ground truth and the predicted maps, the shape of the moving average curve at the local minima can be somewhat flat (esp. for O/E and Z-score); this can lead to a local minimum shifting over a pixel or two. We believe this is within the natural tolerance of TAD calling.

*3. I am not sure I agree with the authors that the goal should be to produce photorealistic images. In my opinion, it is more important to reveal biological insights which are measured by metrics like loop and TAD calling. MSE is obviously not a perfect objective function, but, as demonstrated in the authors' responses to the first review, the corresponding MSE-only model clearly outperforms the proposed model (MSE+GAN). Thus, it is unclear why the focus should be on "perception" that cannot be quantified and is less biologically relevant and quite possibly noisier.*

Knowing that our collaborators in the 3D genomics field do carefully visually inspect and try to reason about structures in Hi-C maps, we feel that there is indeed value to produce maps that look like real Hi-C for biologists, i.e. this is a biologically relevant aspect of the model. We also showed in the previous response that the GAN discrimination score can in fact quantify how “realistic” the image is. However, we accept the reviewer’s point and now present Epiphany as producing two different models, one using MSE+GAN for visual inspection and one using MSE-only for slightly more accurate performance on downstream tasks. We also spend more time explaining these trade-offs in the text.

*4. For the 42 loci tested in Figure R12, the author mentions that Epiphany prediction is "slightly better" when compared to Akita. However, it is difficult to tell the difference. We are not sure if Epiphany is capable of producing cell type-specific prediction. Also, for cell type-specific prediction, it seems that the authors had to actually train using MSE loss because of the lower sequencing depth of the H1 data, similar to longer range interactions.  This is consistent with the concern that GAN loss introduced artificial noise which further undermined cell type-specific prediction performance.*

While Epiphany performs “slightly better” than Akita in terms of the number of test regions on which its correlation is higher, we have amended this statement to say that the methods perform statistically equivalently on the 42 loci based on one-sided Wilcoxon signed rank tests: p-values are not significant in either direction for GM12878 comparing Epiphany (MSE+GAN) with Akita; for H1ESC (Micro-C data), there is a trend of better performance for Epiphany (MSE-only) over Akita (*p* < 0.0894). These results are shown in **Supplementary Fig. S7**; we have added significance tests.

We have included additional data to show “Epiphany is capable of producing cell-type-specific predictions'' in several ways. First, we have added an additional Epiphany analysis where we make predictions in a tissue type from ENCODE, left heart ventricle, where both 1D epigenomic inputs and ground truth intact Hi-C are available (**Supplementary Fig. S9**). Encouragingly, overall performance is comparable to performance on held-out cell line data.

Next, we show that Epiphany better generalizes to a completely held-out cell type than Akita, namely K562 cells. (We did not do this comparison previously because Akita is not meant to be used in this way – it cannot generalize beyond the training cell types – but it seems important to explicitly demonstrate this point.) Here we use 1D inputs in the held-out cell type for Epiphany predictions, and we take the average of Akita’s predictions for training cell types as its predictions (the only possible way to use Akita in a new cell type). These results are shown in **Supplementary Fig. S10**. We split the 42 test regions into *conserved regions* with consistent 3D structure between K562 and the average of the 5 training cell types, and *non-conserved regions* with different structure between K562 and the average of the training cell types. Across both conserved and non-conserved test regions, Epiphany showed consistent performance with ground truth. The average Pearson correlation between Epiphany prediction and ground truth was 0.5227 on conserved regions as compared to 0.5706 on non-conserved regions, and only 5 regions had Pearson correlations lower than 0.4 among all the test regions. By contrast, while Akita tended to predict accurate structures on the conserved regions, it had difficulty with quite a few of the non-conserved regions, suggesting a failure to generalize to the new cell type.

**Related to Figure S10 (revised manuscript)**. Epiphany and Akita prediction on test regions (chr3, 11, 17) on K562. **(A)** Conserved regions. Top row: averaged Akita prediction of 5 cell types (GM12878, H1-hESC, IMR90, HCT116, HFF) on test regions. Middle row: ground truth contact map on K562. Bottom row: Epiphany prediction on K562. Pearson correlations between Epiphany prediction and ground truth are on the top. **(B)** Non-conserved regions. Rows as in **(A)**.

Finally, for a more global assessment of Epiphany’s generalization ability, we restricted to regions that are less conserved between cell types (e.g. different TAD locations, large number of differential interactions) and showed that Epiphany’s prediction performance on these regions is as strong as for conserved structures. Specifically, we used HiCExplorer to identify the conserved vs. non-conserved regions between K562 and HCT116 and calculated the Pearson correlations of the prediction vs. ground truth on the top 25 conserved and non-conserved regions. Here, the Epiphany model was trained on GM12878 and tested on K562 and HCT116. Conserved and non-conserved regions were identified and ranked by the p-value from Wilcoxon rank-sum test in the hicDifferentialTAD function from HiCExplorer. The distribution of Pearson correlations is shown in **Supplementary Fig. S12** and shows similar performance between conserved and non-conserved regions. Example comparisons of non-conserved and conserved regions are shown in **Supplementary Fig.** **S12B** and **S12C**, respectively. The full table of HiCExplorer conserved and non-conserved regions is attached in the **Supplementary Tables**.

*5. Based on the authors' results, GAN loss does not perform well on long-range loops: the authors provide an explanation related to the lower number of reads at longer distances; again this argues in favor of the denoising properties on MSE. This also raises another concern: what is the minimum sequencing depth required for GAN loss to perform well in terms of loop calling at a given distance? The fact that for the H1 cell line the authors had to resort to using the MSE loss is additional evidence that the MSE model is better.* 

To clarify the point we made in the previous response: we do not believe the GAN loss is adding “extra noise” at longer range due to lower read coverage in the results shown; also please note that our observed/expected target values correct for distance-dependent coverage bias, so there is not an issue of lower number of reads at longer distances per se. Rather, there are fewer true interactions at longer ranges. In this setting of sparser true positives, the highly smoothed (“blurry”) MSE-only predictions will have higher recall. In our revision, we have clarified this point, i.e. we have explained the trade-off between “higher resolution” (non-blurry) and more realistic predictions that are useful for visual inspection vs. blurry MSE-only predictions that have somewhat higher recall for loop calling, especially at longer distances where true interactions are sparse. We recommend the MSE+GAN version of Epiphany for the visual inspection task and the MSE-only version for the downstream loop-calling task.

With respect to the reviewer’s question about dependency on coverage, all models so far (e.g. Akita, deepC) have only been trained on the highest resolution Hi-C data sets; all models would be expected to degrade in performance with lower sequencing depth (and hence noisier) Hi-C maps. As the reviewer suggests, we do expect that when training on highly noisy Hi-C data, the GAN component of the model would be less useful for learning perceptually realistic contact maps. We have added a statement to the H1ESC results section noting that we used MSE-only in this lower data quality setting for this reason.

*Minor comments:
1.	To convert predicted Hi-C matrix to read pairs for loop calling, the authors could first convert the intensity s between location i and j to integers int(S) and generate int(S) number of lines of i and j pseudo interaction reads in the read pair files.*

Thank you for this suggestion. However, our observed/expected (O/E) target values allow a simple interpretation of the predicted values in terms of significance of the interactions (and these should not be viewed as read pairs, which would be closer to “O” rather than “O/E”).

*2.	For comparison between CNN and LSTM, the author could use a larger CNN input range. We suggest either removing Figure 5C or run the comparison between CNN and LSTM with the same input window.*

We have removed the previous **Fig. 5C**.

*3.	Can the author provide the code they used to generate the quantitative evaluation figures?*

We will upload all the code to the GitHub page.

*4.	Human gene name, e.g. SOX17, should be all capital letters. Errors include Sox17.*

We have fixed human vs. mouse gene conventions in all figures.

**Third round of review**

**Reviewer 1**

The authors have addressed the remaining comments. I think it is a good idea to present both models and let the user choose according to the specific application.
